# Modulation of the Kynurenine Pathway: A New Approach for Treating Neurodegeneration

**DOI:** 10.3390/life16020266

**Published:** 2026-02-03

**Authors:** Julia K. Banaszkiewicz, Anna Kukiełka, Elżbieta Kudyk, Łucja J. Walczak, Katarzyna Wicha-Komsta, Mariola Herbet, Iwona Piątkowska-Chmiel, Grzegorz Nowicki, Carmen E. Mielnik, Tomasz Kocki

**Affiliations:** 1 Chair and Department of Toxicology, Faculty of Pharmacy, Medical University of Lublin, Jaczewskiego 8b Street, 20-090 Lublin, Poland; juliabanaszkiewicz00@gmail.com (J.K.B.); 61695@umlub.edu.pl (A.K.); elakudyk@wp.pl (E.K.); lucja.walczak@umlub.edu.pl (Ł.J.W.); mariola.herbet@umlub.edu.pl (M.H.); iwona.piatkowska-chmiel@umlub.edu.pl (I.P.-C.); 2 Institute of Medical Sciences, Faculty of Medicine, The John Paul II Catholic University of Lublin, Konstantynów 1H Street, 20-708 Lublin, Poland; katarzyna.wicha-komsta@kul.pl; 3 Department of Family and Geriatric Nursing, Faculty of Health Sciences, Medical University of Lublin, 20-090 Lublin, Poland; grzegorz.nowicki@umlub.edu.pl; 4 County Health Center in Opole Lubelskie, Przemysłowa 4a, 24-300 Opole Lubelskie, Poland

**Keywords:** neurodegenerative diseases, kynurenic acid, kynurenine pathway

## Abstract

Neurodegenerative diseases, such as Parkinson’s and Alzheimer’s, are becoming an increasingly serious challenge for modern medicine because of the significant increase in incidence and the narrow range of effective therapeutic strategies. In recent years, the kynurenine pathway, which is one of the main pathways of tryptophan metabolism, responsible for the synthesis of products that act oppositely in the CNS including neurotoxic (quinolinic acid) and neuroprotective products, has gained increasing recognition as a potential therapeutic target. Abnormalities in the production of these metabolites, causing a disruption of homeostasis in the CNS, often lead to the development of inflammation, which can cause oxidative stress or neuronal death. This paper aims to discuss strategies useful in modulation of the kynurenine pathway, based on increasing the production of neuroprotective metabolites and reducing the synthesis of neurotoxic compounds, as well as to outline the progress in preclinical and clinical studies and the challenges encountered in these studies, among others, in the search for new KP inhibitors. The pharmacological (IDO and KMO inhibitors) and non-pharmacological (physical activity, diet) strategies are discussed, as well as new approaches from combination and targeted therapies. Together with the results of preclinical studies, they demonstrate the high utility of this target in the treatment of neurodegeneration. Despite its promising activity, further key studies are needed to fully understand the mechanisms involved in metabolism, which may translate into increased efficacy of developed therapies in the future.

## 1. Introduction

Neurodegeneration is one of the most serious challenges of modern medicine and neurological sciences. It is a process of progressive damage and death of nerve cells, leading to gradual loss of cognitive, motor, and emotional functions [1,2]. Although the most well-known neurodegenerative diseases, such as Alzheimer’s disease, Parkinson’s disease, and amyotrophic lateral sclerosis (ALS), differ in clinical picture, pathogenesis and specific degenerative alterations in different regions of the CNS, they share several converging pathogenic mechanisms, including chronic neuroinflammation mediated by glial activation, oxidative stress resulting from impaired redox homeostasis, dysruption of intracellular calcium signaling, mitochondrial bioenergetic failure, and misfolding and aggregation of disease-specific proteins. Alterations of the immune system constitute an integral component of neurodegenerative processes. Increasing evidence indicates that chronic immune dysregulation, including persistent low-grade neuroinflammation, microglial activation, and altered cytokine signaling, contributes significantly to neuronal dysfunction and disease progression. In parallel, researchers are increasingly focusing on the connections between metabolic diseases, such as type 2 diabetes, and neurodegeneration. In this context, the kynurenine pathway (KP) has gained growing attention, as it plays a pivotal role in immune–metabolic crosstalk, regulating inflammatory processes, oxidative stress, and the balance between neurotoxic and neuroprotective metabolites in the central nervous system (CNS) [3].

Chronic neuroinflammation is one of the processes leading to neurodegeneration. In normal conditions, neuroinflammation is an important element of the brain’s defense against various types of damage, infections, toxins, or metabolic changes [3,4]. The main role in defense is played by microglial cells and astrocytes. In a physiological state, microglia remove damaged cells, cleanse the environment from toxins, support tissue repair, and regulate synaptic plasticity. Constant activation by the damage mentioned above also disturbs homeostasis and transforms microglial cells into a chronic, pathological state. Microglia activation is also associated with activation of the KP [5]. Enzymes such as indoleamine 2,3-dioxygenase (IDO) and tryptophan 2,3-dioxygenase (TDO), activated in the response to inflammatory stimuli, convert tryptophan (TRP) to neurotoxic metabolites, including quinolinic acid (QUIN) and 3-hydroxykynurenine (3-HK), thus exacerbating neuronal damage. Microglia transform into a pro-inflammatory state, secreting cytokines (interleukin (IL)-1β, tumor necrosis factor (TNF)-α, IL-6), and reactive oxygen and nitrogen species (ROS and RNS) [6]. They affect the relaxation of the blood–brain barrier (BBB), which toxins, pathogens, and inflammatory cells pass from the blood into the CNS. This leads to disturbances in ionic homeostasis and neurotransmission, which affects the degree, severity, and type of neurodegenerative disease [3].

In inflammatory conditions, astrocytes change their phenotype to a neurotoxic one, and begin to produce mediators of inflammations, thereby inhibiting the regeneration of CNS cells [7]. Importantly, QUIN acts not only as a direct neurotoxicant but also as an activator of the NOD-like receptor pyrin domain-containing 3 (NLRP3) inflammasome, resulting in a looping inflammatory response in the brain. The neurotoxic phenotype is characterized primarily by increased production of chemokines and inflammatory cytokines, intensifying local inflammation. Astrocytes lose also their ability to remove glutamate, leading to excitotoxicity [8]. QUIN, as an N-methyl-D-aspartate (NMDA) receptor agonist, enhances excitotoxicity by increasing the influx of calcium ions into the neuron, which contributes to cell death and neurotransmission disorders. In turn, a decrease in the concentration of kynurenic acid (KYNA), which has neuroprotective properties, weakens the brain’s defense mechanisms.

Considering neuroinflammation, attention should be paid to the NLRP3 inflammasome: a protein complex present in microglia, playing an important role in the initiation and promotion of the inflammatory response in the CNS [9,10]. Its activation occurs in two stages: in the first phase (priming), infectious agents or cell damage signals (pathogen-associated molecular patterns (PAMP), damage-associated molecular patterns (DAMP) activate the Toll-like receptor (TLR) and the NF-κB pathway, which leads to increased expression of its components, as well as inactive precursors of proinflammatory cytokines—pro-IL-1β and pro-IL-18. In the second phase, secondary stimuli such as oxidative stress, extracellular ATP, or amyloid β deposits lead to NLRP3 oligomerization, recruitment of the caspase recruitment domain (ASC) adaptor, and activation of caspase-1, which converts cytokine precursors into active forms: IL-1β and IL-18. The effect is the development of chronic inflammation, which intensifies neurodegeneration. In Alzheimer’s disease, NLRP3 activation by β-amyloid deposits leads to excessive activation of microglia, deepening neuronal damage, and cognitive impairment. Studies indicate that silencing NLRP3 activity can induce a neuroprotective effect, making the inflammasome a potential therapeutic target in neurodegenerative diseases. More and more attention is also being paid to disorders of the KP, which plays an important role in TRP metabolism [11]. This pathway is activated in many neurodegenerative diseases and accompanies impairments in them.

In the neuroinflammatory state, the balance shifts towards neurotoxic metabolites such as 3-HK and QUIN, at the expense of KYNA, which has a neuroprotective effect [12]. Such changes increase oxidative stress, mitochondrial damage, and excitotoxicity. Oxidative stress promotes the occurrence and progression of neurodegenerative diseases [13]. The brain is an organ with high oxygen consumption and limited regenerative capacity; therefore, it is very susceptible to damage caused by free oxygen and nitrogen radicals. As a result of neurodegenerative diseases, the disruption of homeostasis in the CNS damages cellular structures and causes the death of neurons. Oxidative stress not only intensifies inflammatory processes and disrupts the functioning of mitochondria but also promotes the aggregation of pathological proteins.

Abnormal protein aggregation is another significant pathophysiological mechanism underlying many neurodegenerative diseases [14]. Interestingly, disorders of the KP also correlate with this mechanism. In Alzheimer’s disease, QUIN levels increase in the moderate stages of the disease, reaching a maximum concentration at the peak of the disease. Its presence may enhance β-amyloid aggregation and hyperphosphorylation of tau protein, which forms neurofibrillary tangles inside neurons. In physiology, neurons have mechanisms that maintain balance, ensuring proper folding of proteins, and eliminating dysfunctional ones [15].

Chaperones, proteasomes, and autophagy are their main reasons [16]. Consequently, CNS disorders dysregulate proteostasis, causing abnormal proteins and insoluble protein aggregates to accumulate within neurons and the extracellular space. In Alzheimer’s disease, a characteristic phenomenon is the deposition of amyloid-β in the form of senile plaques and hyperphosphorylated tau protein, which forms neurofibrillary tangles inside neurons. In Parkinson’s disease, the key role is played by the aggregation of α-synuclein and the formation of so-called Lewy bodies, while in ALS or Huntington’s disease, aggregates of other, specific proteins (e.g., huntingtin).

Pathological aggregation is toxic to neurons, causing neuroinflammation and increasing oxidative stress [15]. Axonal transport is inhibited, and mitochondrial function is impaired. Additionally, pathological proteins can trigger other molecules and propagate incorrect folding, which deepens neurodegeneration. It is noteworthy that disorders associated with the KP pathway correlate with amyloid-β and tau pathology [17]. In Alzheimer’s disease, for example, QUIN concentrations peak in the middle stages of the disease and then decline in the late stages of the disease.

Microglia, which physiologically has protective functions by removing dead cells, may also facilitate the pathogenesis of these diseases, promoting tissue regeneration, and maintaining synaptic homeostasis [18,19]. However, in response to the accumulation of pathological proteins, such as the previously mentioned β-amyloid, hyperphosphorylated tau, or α-synuclein, microglia become excessively activated and adopted a pro-inflammatory phenotype (M1). In Alzheimer’s disease, microglia activation in response to β-amyloid leads to its phagocytosis, but with continuous exposure, these cells lose the ability to remove it effectively and become a source of neurotoxic mediators themselves. In addition to cytokines and ROS, microglia can also produce QUIN, exacerbating the toxic effect on neurons. This additional mechanism may partially explain the observed progression of neurodegenerative changes in diseases such as Alzheimer’s and Parkinson’s disease.

In Parkinson’s disease, similar mechanisms of microglial activation are triggered by α-synuclein aggregates, contributing to the degeneration of dopaminergic neurons. Moreover, chronically activated microglia generate excessive amounts of QUIN, which, as previously noted, is linked to the progression of neurodegenerative disease [17].

It is important to note that neurodegeneration is not a single process confined to neurons but it is frequently accompanied by complex interactions with glial cells, particularly microglia and astrocytes [20]. Activated glial cells release proinflammatory cytokines and ROS, which exacerbate neuronal damage and perpetuate neuroinflammation, creating a vicious cycle. At the cellular level, neurodegeneration is often the result of abnormal protein aggregation, which can lead to impaired neuronal function [21]. The effects of these processes include cellular stress, disruption of cell signaling, and loss of metabolic homeostasis. As a result, neurotransmitter release is impaired, and synaptic plasticity is weakened. This leads to disorganization of the neuronal networks responsible for cognitive, sensory, and motor functions. As the disease progresses, damaged neurons undergo apoptosis, which contributes to the loss of specific brain structures.

The clinical manifestations of neurodegeneration depend on the anatomical regions and neural circuits affected [20]. For example, Alzheimer’s disease is characterized by degeneration of cholinergic neurons, leading to memory impairment, while disorders in dopaminergic neurons in the gray matter characterize Parkinson’s disease, manifesting as tremor or bradykinesia. In addition, it has also been shown that increased QUIN levels correlate with reduced cognitive performance in these patients [17].

Despite intensive research, understanding the exact etiological factors responsible for neurodegeneration remains largely unknown [22]. Changes in the KP can result from both genetic and environmental factors—such as chronic stress, infection, insulin resistance, or metabolic disorders. Excessive activation of the enzyme kynurenine-3-monooxygenase (KMO) shifts metabolism toward QUIN and 3-HK, increasing the brain’s susceptibility to oxidative and inflammatory damage. The influence may be multifactorial, starting from genetic predisposition, through aging, to environmental factors. Understanding the underlying pathophysiology may help in therapies for diseases previously treated only symptomatically, focusing on causal treatment. The study aims to analyze the significance of KP modulation in the treatment of selected neurodegenerative diseases. In addition, the latest therapeutic proposals and challenges related to research on the role of this pathway in the pathogenesis and treatment of these diseases were reviewed.

## 2. The Kynurenine Pathway as a Therapeutic Target in Neurodegenerative Disorders

### 2.1. The Purpose of Searching for New Therapeutic Methods

At present, neurodegenerative diseases such as Alzheimer’s disease, Parkinson’s disease, or ALS remain incurable, and the therapies available on the market are only symptomatic [22,23]. They mainly consist of relieving symptoms, without affecting the recession of the process. Due to the dramatic consequences of these diseases—both for patients, their families, and entire health care systems—the search for new therapeutic methods is one of the key challenges of modern medicine.

Modern therapeutic strategies focus on several major goals [24]. First, scientists focus on processes and their modifications that would slow down and stop the progression of neurodegenerative changes, instead of only alleviating clinical symptoms. Scientists focus on mechanisms such as reducing the deposition of pathological proteins (β-amyloid, tau, α-synuclein), modulating the neuroinflammatory response, reducing oxidative stress, or improving mitochondrial function and supporting neuronal regeneration.

One new and forward-looking strategy is the concept of multi-targeted ligands (MTDLs) [25,26]. These molecules are designed to modulate multiple pathological targets involved in neurodegeneration simultaneously. This is important, given the complex and multifactorial nature of diseases such as Alzheimer’s. Traditional single-drug treatment approaches have shown limited efficacy, failing to take into account interrelated mechanisms such as the above-described protein aggregation, oxidative stress, mitochondrial dysfunction, and nervous system inflammation.

MTDLs combine pharmacophores targeting different mechanisms in a single structure: they can, for example, concurrently inhibit acetylcholinesterase (AChE)—increasing acetylcholine levels and improving cognitive function- and neutralize free radicals through antioxidant activity [27]. Other combinations include anti-inflammatory activity, inhibition of β-secretase (BACE1)- involved in β-amyloid production—or NMDA receptor modulation, affecting neuroprotection. Multidrug therapies carry a fairly high risk of drug–drug interactions and can accumulate side effects [26]. For this reason, MTDLs represent a safer and more targeted solution, as they can cross the BBB, which definitely affects the promising potential of the therapy. Examples of MTDL molecules analyzed in the study are hybrids of donepezil and ferulic acid or curcumin, combining anticholinesterase and antioxidant activity.

The aforementioned molecules targeting AChE and monoamine oxidase (MAO) B simultaneously are important in regulating neurotransmission and oxidative stress [27]. Then some ligands modulate the KP, affecting, for example, the activity of KMO, whose products—such as QUIN- are neurotoxic.

The molecules are being developed primarily to inhibit or slow neurodegeneration by acting on several disease mechanisms in parallel, offering the chance for more effective treatment and greater personalization of therapy [28]. Second, targeted and personalized therapies that are tailored to the individual biological characteristics of the patient are becoming increasingly important [29]. They focus on tailoring therapy to a patient’s individual biological profile—including genetic, epigenetic, and metabolic factors [30]. With neurodegenerative diseases, personalized intervention is based on risk factors, the stage of the disease, or the level of biomarkers, such as the level of KP metabolites. That is made possible by continuing advances in genetics and molecular biomarkers, making it possible to select the most personalized therapy. Third, immunomodulation therapies and strategies based on interference with kynurenine metabolism, the deregulation of which plays an important role in the pathogenesis of many neurodegenerative diseases, are also developing rapidly [31]. With the growing awareness of the function of microglia and the BBB, novel approaches are being developed based on restoring homeostasis within the nervous system and protecting neurons.

Neurodegenerative diseases are difficult to treat because they have a very complex etiology [22,32]. Various processes are involved in their pathogenesis, such as aggregation of pathological proteins (e.g., β-amyloid, tau, α-synuclein), chronic inflammation, mitochondrial dysfunction, disorders of TRP metabolism and the KP, and even environmental and lifestyle factors. Hence, precision medicine will involve studies such as genotyping patients, analyzing their biomarkers (CSF protein levels, profile of pro-inflammatory cytokines, metabolites of the KP), regular monitoring of response to treatment, and finally selecting drug and non-pharmacological therapies. An example of the use of biomarker-based therapy would be the testing of serum QUIN levels, which can be reduced with appropriate KMO inhibitors that are neuroprotective [32].

In this context, KP becomes a therapeutic target to treat neurodegenerative diseases. KP metabolites also exhibit both neurotoxic and neuroprotective effects. Targeting enzymes of this pathway (e.g., kynurenine 3-monooxygenase, or KAT) as part of a multi-target or individualized therapy strategy may offer new opportunities to modulate disease progression [33].

### 2.2. Introduction to TRP Metabolism and the KP

TRP is one of the essential amino acids, playing a key role as a precursor of many biologically active compounds and in protein synthesis. In the human body, its metabolism consists of three pathways: the kynurenine, serotonin, and indole [34]. Among these, the KP is responsible for the breakdown of more than 95% of the TRP pool in the body.

**Figure 1:**
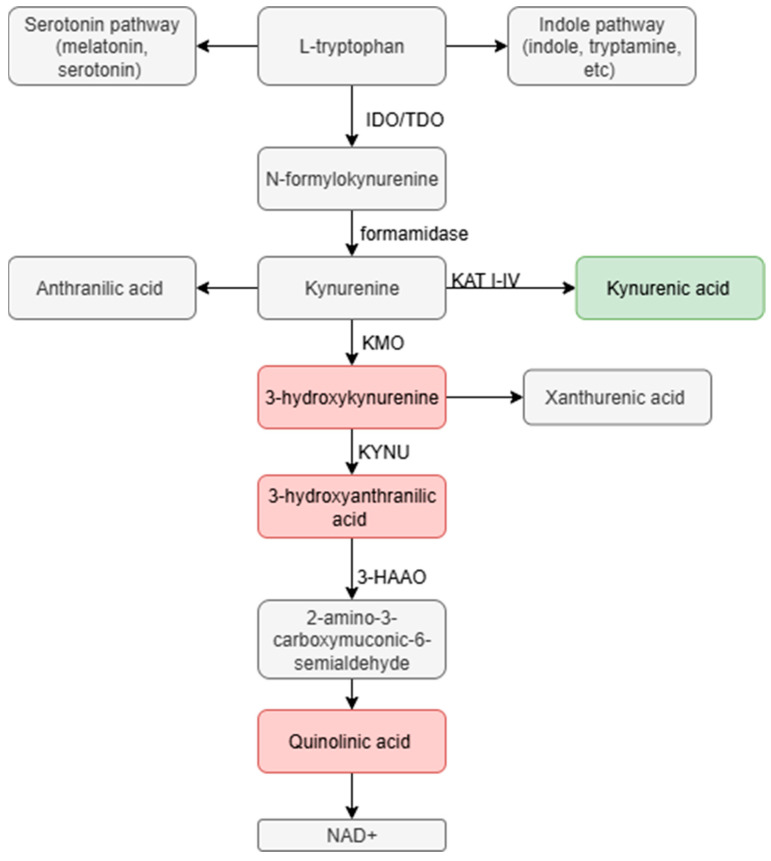
Tryptophan metabolism via the kynurenine pathway. Metabolite with neuroprotective effects is indicated in green, while those with predominant neurotoxic effects are indicated in red. KAT-kynurenine aminotransferases; KMO-kynurenine-3-monooxygenase; KYNU-kynureninase; 3-HAAO-3-Hydroxyanthranilate 3,4-dioxygenase.

The KP begins with the conversion of TRP into N-formylkynurenine (Figure 1). The enzymes that catalyze the reaction are: IDO1/IDO2 or TDO [35]. Many tissues contain IDO, which is induced by proinflammatory cytokines such as interferon (IFN)-γ, while the liver mainly produces TDO, and glucocorticoid and TRP levels regulate it. The subsequent steps in the pathway lead to the formation of several metabolites, including KYN, KYNA, 3-HK, QUIN, and others, which differ in their biological activity-exerting neuroprotective and neurotoxic effects [36]. This makes the KP an important element in the regulation of nervous system homeostasis. Moreover, the final product of KP is NAD+ (nicotinamide adenine dinucleotide), which is crucial in cellular energy metabolism and redox processes. In this way, this pathway connects immune, metabolic, and neuroregulatory functions, and its disruption can lead to disorders related to inflammation, oxidative stress, and neurodegeneration [37].

The steps of the KP include many metabolites with diverse effects on the nervous, immune, and metabolic systems [38]. Four of them play a special role because of their biological activity: KYN, KYNA, 3-HK, and QUIN. Disturbances in these metabolites are associated with the pathogenesis of many neurodegenerative diseases, conditioning the state of neuroimmune homeostasis. In the first stage of the KP, IDO or TDO enzymes convert TRP to form KYN. KYN itself does not exert direct neuroactive effects; however, it is a key stage in TRP metabolism. Metabolites with opposing properties are formed from KYN: neuroprotective KYNA via the astrocytic pathway, and neurotoxic 3-HK and QUIN via the microglial and monocytic pathway [39].

KYN can also activate the aryl hydrocarbon receptor (AhR)—a transcription factor affecting the development and function of the immune system, metabolism, and cell differentiation [40]. Activation of AhR by KYN is associated with the suppression of inflammatory reactions and the induction of immune tolerance. Increased levels of KYN in serum and cerebrospinal fluid are observed in neurodegenerative pathologies such as multiple sclerosis and Parkinson’s disease [38].

Astrocytes mainly produce the aforementioned KYNA. It acts, among other things, as an endogenous non-competitive antagonist of the NMDA receptor, which reduces excessive glutamatergic activation and protects against oxidative stress and, consequently, against excitotoxicity [41]. KYNA blocks NMDA receptors and limits the influx of Ca^2+^ ions into the interior of neurons. Binding mainly caused glutamate antagonism of KYNA against NMDA receptors with the glutamate site on the GluN2 subunit [42]. However, it also blocks the co-agonist glycine-B site on the GluN1 subunit. Thanks to this antagonism, KYNA has strong neuroprotective properties, protecting mitochondria, and inhibiting the activation of apoptotic pathways.

KYNA also antagonizes α7nAChR receptors, which are present on neurons and glial cells [43]. Their blocking process indirectly reduces the release of glutamate by presynaptic nerve endings. In high concentrations, KYNA can also block other glutamate receptors (AMPA, kainate), although its binding to the receptor is much weaker than in the above-mentioned NMDA and α7nAChR [43]. KYNA can also affect astrocyte microglia, reducing their ability to secrete glutamate under conditions of stress and inflammation.

Other important metabolites of the KP, such as QUIN and 3-HK, are mainly synthesized in microglia and monocytic cells. These metabolites act in opposition to KYNA, promoting neurotoxic processes within the CNS. As long as neurotoxic metabolites remain in balance with KYNA, neurophysiological homeostasis is preserved, but disturbance of this ratio is associated with the progression of neurodegenerative diseases [44,45]. In contrast to KYNA, QUIN is a potent endogenous agonist of NMDA receptors, which belong to the ionotropic glutamate receptor family [12,46]. Increased synthesis of QUIN leads to excessive calcium influx into neurons, causing mitochondrial damage and overproduction of ROS, which results in oxidative stress and ultimately triggers apoptosis. In addition, QUIN enhances glutamate release from astrocytes, initiating an excitotoxic loop- a self-perpetuating mechanism in which neuronal death caused by excessive glutamate further amplifies glutamate release and exacerbates neurotoxicity [12]. Additionally, QUIN promotes lipid peroxidation of cell membranes through ROS generation.

3-HK is another highly reactive metabolite of KP, with a strong oxidative potential. Although it does not directly act on glutamate receptors, it serves as a donor of ROS and undergoes autoxidation [47]. ROS react with various components of proteins, including thiol groups (-SH) on cysteine, which are crucial for the structural and functional integrity of proteins [48]. Sulfhydryl groups on cysteine provide a site where it can react with ROS, resulting in modifications such as disulfide formation or oxidation of sulfhydryl groups. This leads to changes in protein conformation, disrupting their function. Such structural changes can disrupt the enzymatic activity of proteins involved in metabolism, cell signaling, or cell cycle control. Carbonylation is another process that is a consequence of ROS accumulation, in which carbonyl groups are added to proteins, leading to their denaturation and loss of function [48]. This negatively affects both signaling and structural integrity of neurons. 3-HK is able to damage proteins involved in signaling pathways, such as protein kinases, receptors, and other protein elements involved in signal transduction. Oxidative modifications of these proteins can lead to disturbances in their activity, which contributes to the pathogenesis of many neurodegenerative diseases [49]. In addition, ROS can lead to DNA strand breaks. Damage can occur in both strands of DNA, disrupting proper replication and gene transcription, leading to loss of genetic information or cellular dysfunction. DNA damage induced by 3-HK increases neuronal susceptibility to further oxidative stress, thereby accelerating degeneration and contributing to the development of neurodegenerative diseases. Disturbance in the ratio of QUIN and 3-HK to KYNA results in the so-called neurotoxic KP phenotype, observed in many neuroinflammatory and neurodegenerative diseases [50]. Activation of microglia and inflammatory cytokines (e.g., IFN-γ, TNF-α) promotes the expression of enzymes such as KMO, which directs the metabolism of KYN towards 3-HK and QUIN [51]. Metabolite imbalances in the KP are a key pathological mechanism observed in many neurodegenerative and neuroinflammatory diseases, such as Alzheimer’s disease, Parkinson’s disease, or multiple sclerosis.

In healthy conditions, this pathway maintains a stable balance between metabolites with neuroprotective and neurotoxic properties [38]. However, when chronic inflammation occurs in the CNS, enzymatic activity shifts towards neurotoxic products. Pro-inflammatory cytokines such as IFN-γ and TNF-α activate microglial cells, which are the main source of enzymes that metabolize KYN to 3-HK and QUIN.

A key enzyme driving this shift is KMO, whose expression is significantly upregulated in response to inflammatory signals.

This upregulation enhances the conversion of KYN to 3-HK, a pro-oxidant metabolite generating ROS, and subsequently to QUIN, an NMDA receptor agonist responsible for excitotoxicity [52]. In the meantime, the activity of enzymes responsible for the production of KYNA, such as KATs, decreases, leading to reduced synthesis of this neuroprotective metabolite. KYNA acts as an antagonist of NMDA and α7nAChR receptors, thereby inhibiting excessive glutamatergic excitation and oxidative stress. A reduction in KYNA levels diminishes this protective effect, enhancing the toxic actions of QUIN.

## 3. Modulation of the KP

In recent years, there has been increased interest in the topic of the KP and its disorders, as a potential cause of the development of neurodegenerative diseases, such as multiple sclerosis, Huntington’s disease, Parkinson’s disease, and Alzheimer’s disease [13,53]. An imbalance between the production of neurotoxic (QUIN) and neuroprotective metabolites (KYNA) inside the pathway may promote the induction of oxidative stress, chronic inflammation, and excitotoxicity and thus lead to disruption of homeostasis in the CNS, contributing to the progression of these diseases.

Enzymes such as KMO, IDO, and KAT, that regulate TRP metabolism, are potential therapeutic targets [53]. In addition, the KP, through the activity of the IDO enzyme, is an important element involved in the regulation of the immune response and activation of inflammation, compounding its importance in neurodegeneration.

Therefore, the pathway modulation strategies aimed at shifting the balance towards products with protective properties and reducing the amount of toxic metabolites that can potentially slow or inhibit neurodegenerative processes became a promising line of research seeking new therapies useful in treating diseases with this background [54]. Nevertheless, a successful modulation of the pathway in clinical application requires overcoming several limitations, such as the problem of drug delivery to the CNS and the emergence of complex immune processes associated with the pathway.

### 3.1. Pathway Modulation Strategies

Transformations in the KP, which is the main pathway of TRP catabolism, lead to the production of several neuroactive intermediate products [55,56]. Some of these metabolites show neuroprotective effects, while some are characterized by strong neurotoxicity in the CNS. These characteristics made KP a potential therapeutic target useful in the treatment of neurodegenerative diseases, and these possibilities have prompted researchers to search for new strategies aimed at modulating it, which would result in reducing the production of harmful metabolites and enhancing the synthesis of neuroprotective ones in the brain. There are many options and pathways for modulating the KP, with both pharmacological and non-pharmacological methods gaining increasing recognition among researchers and becoming the focus of many studies evaluating their effects on TRP metabolism, an effect that often translates into improved outcomes in animal models of neurodegenerative diseases [57,58]. The most widely used methods certainly include the classic pathway enzyme inhibitors TDO, IDO1, IDO2, KMO, and KATII, which are by far the most popular in preclinical studies. Despite their proven efficacy, other equally effective and safe strategies are also being considered, based on lifestyle and dietary changes, probiotic supplementation, and the use of pathway modulators at the cofactor (vitamin B6) and receptor levels (NMDA).

#### 3.1.1. Vitamins B6 and B2

One of the factors reported by many scientific sources to be crucial in modulating the KP and affecting the production of the neuroprotective metabolite, KYNA, is the supplementation of pyridoxine (vitamin B6), which plays an important role in the normal functioning of the nervous system [59,60,61]. It is responsible for mediating the synthesis of neurotransmitters or regulating homocysteine metabolism and exhibits antioxidant and anti-inflammatory effects. It is also a cofactor of more than 140 enzymes, making it essential for the normal course of many reactions in the human body. Low vitamin B6 status are sometimes observed in patients with Parkinson’s and Alzheimer’s disease [62,63,64].

The proper functioning of key enzymes of the pathway, such as KYNU and KATs, is dependent on the presence of pyridoxal 5′-phosphate (PLP), the active form of vitamin B6 [65]. Thanks to PLP, KAT enzymes can actively convert KYN to KYNA, while PLP deficiency caused by low vitamin B6 status in the body results in excessive accumulation of neurotoxic products and reduced KYNA levels. In a study by Midttun et al. in patients with cardiovascular disease, it was observed that higher PLP levels were associated with lower plasma of 3-HK levels. In addition, a negative correlation was observed between PLP and the ratio of 3-HK to KYNA [66]. Daily supplementation of pyridoxine hydrochloride (40 mg/day) for one month led to an approximately eightfold increase in plasma PLP levels and a reduction in 3-HK levels from 29.5 nmol/L to 20.2 nmol/L, which also resulted in a reduction in the 3-HK: KYNA ratio. This indicates that low levels of vitamin B6 in the body are associated with significantly higher production of neurotoxic metabolites of the KP. In clinical practice, PLP measurement remains the primary marker of vitamin B6 deficiency. However, measurement of 3-HK could in the future support the assessment of vitamin B6 metabolism disorders, especially in high-risk populations. Furthermore, given the strong association of vitamin B6 with effects on the nervous system, it is necessary to analyze more precise mechanisms that would also link its levels to other changes in the KP pathway. In contrast, a study by Yeh and Brown in humans, guinea pigs, rats, and hamsters after oral TRP loading showed that vitamin B6 deficiency caused a significant increase in the intensity of urinary excretion of xanthurenic acid (XA), KYN, and 3-HK in all four species studied [67]. In the absence of vitamin B6 deficiency, significantly lower urinary excretion of products of the KP was found after TRP supplementation. The prospective Boston Puerto Rican Health Study associated low PLP values (<43 nmol/L) with a 2.5-fold higher risk of significant (≥1 SD) cognitive impairment in study patients over 2 years [68]. Furthermore, the results of the CARDIA study showed a correlation between higher vitamin B6 intake in young people with better performance on psychomotor speed tests in older age, which may translate into a significantly lower risk of developing dementia in later life [69]. Many studies and research support the fact that vitamin B6 plays an important role in the normal functioning of the nervous system and in shifting the balance of the KP towards the production of neuroprotective metabolites, which is important in the context of modulation of this pathway and therapy of neurodegenerative disorders [63,70].

Vitamin B2 (riboflavin) is a key cofactor that plays comparable role in TRP metabolism to that of vitamin B6. Its active coenzymatic forms, flavin adenine dinucleotide (FAD) and flavin mononucleotide (FMN), are factors responsible for regulating the action of several KP enzymes [71]. One of the enzymes, which needs the presence of FAD for proper functioning, is the KMO enzyme, which is thought to convert KYN to 3-HK. Vitamin B2 deficiency can lead to reduced KMO activity resulting in excessive KYN accumulation, leading to an imbalance between the protective and toxic products of the pathway. On the contrary, excessive riboflavin is not beneficial, as it may enhance the formation of HAA and XA, which, in excess, can have detrimental effects on the CNS. Interestingly, vitamins B2 and B6 can complement each other in their effects on certain metabolites, such as XA. However, at riboflavin concentrations exceeding the population median (13 nmol/L), the relationships between riboflavin concentrations and pathway metabolites level reached a plateau phase, suggesting that increased dietary intake of vitamin B2 may be unnecessary in individuals with normal nutritional status and will not result in benefits in pathway modulation.

#### 3.1.2. Probiotic Supplementation and the Gut–Brain Axis

In recent years, in the context of research into the pathogenesis of neurological diseases, one of the main subjects of research has become the involvement of the gut–brain axis, which is a system that connects the gut microbiota to the CNS through neural (the vagus nerve) or immunological pathways [72,73]. In many neurodegenerative diseases such as Alzheimer’s and Parkinson’s, gut dysbiosis promotes the development and further progression of neurodegenerative processes.

Mice lacking gut microbiota exhibit delayed myelination and abnormal neurogenesis in the hippocampus, which was partially restored following microbiota colonization [72,74]. Accordingly, supplementation with probiotics containing well-characterized bacterial strains studied for this purpose represents a promising strategy for KP modulation [75].

Probiotics may modulate the KP by influencing TRP metabolism potentially enhancing the conversion of compounds such as KYN into neuroactive metabolites, such as KYNA [76,77]. Several studies have been conducted how specific probiotic bacterial strains modulate the KP. In experiments examining a strain of *Lactobacillus reuteri*, it was observed that these bacteria extensively take up KYN and metabolize it to KYNA, which may promote beneficial regulation of TRP metabolism in the gut–brain axis and alleviate neuropsychiatric disorders.

In contrast, studies analyzing the properties of strains of *Lactobacillus casei*, *Pseudomonas fluorescens*, *Escherichia coli*, *Streptococcus salivarius*, and *Streptococcus thermophilus* revealed that each of these species has different effects on the activity of enzymes of the KP [78]. For example, the *Lactobacillus casei* strain increased both KYN levels and its conversion to KYNA, while *Streptococcus thermophilus* showed no comparable effect. Therefore, when constructing probiotic-based therapies targeting modification of the TRP metabolism pathway, it is crucial to select the right strains and take into account their interrelationships within the gut microbiota. This, in turn, can translate into real benefits for brain function and mental health.

Reviews of studies evaluating the effectiveness of probiotic and prebiotic therapies in modulating KP showed that their use had a significant effect on at least one product of the pathway [79]. A meta-analysis by Purton et al. showed that the introduction of pre- and probiotic supplementation was associated with a decrease in KYN levels and a reduction in the KYN/TRP ratio in most subjects. Large differences in the methodology of the analyses conducted, such as the different timing of the studies, different doses or strains used, and differences in the participant populations, limit the ability to make firm conclusions about the effectiveness of the therapy. There is a need for further studies, more methodologically standardized and conducted in populations with neurodegenerative disorders. Reports are showing that synbiotic therapies combining pre- and probiotics may have a much stronger biological effect, but this needs to be confirmed.

*Bifidobacterium infantis* strains commonly used as active ingredients in probiotics also modulate the KP by shifting the balance toward neuroprotection [80,81]. A similar effect is demonstrated by the probiotic *Lactobacillus plantarum*, which led to an effective reduction in KYN levels, resulting in a significant improvement in cognitive function. Combination therapies including popular strains of probiotic bacteria have a beneficial effect: *Bifidobacterium lactis*, *Bifidobacterium bifidum*, *Lactobacillus casei* and *Lactobacillus acidophilus* have been shown to improve the integrity of the BBB. Furthermore, *Lactobacillus casei Shirota* positively affecting the gut microbiome alleviates certain symptoms in Parkinson’s disease patients, supporting the idea that gut health plays an important role in maintaining normal CNS function.

Treatment with *Bifidobacterium longum* strain *CCFM1077* in rats reduced neurotoxic QUIN levels and alleviated autism symptoms [82]. Intestinal dysbiosis, inflammation and disruption of the intestinal barrier caused by chronic ketamine administration lead to the inflammatory processes in neural tissue and activation of the KP manifested by decrease in TRP and QUIN levels along with increased KYN and KYNA concentrations [83]. As a result of these changes, memory and cognitive function are impaired in the test animals. Treatment with inulin, a prebiotic substance, effectively reversed these processes by increasing colonization with beneficial bacteria such as *Turicibacter* while reducing potentially harmful strains like *Desulfovibrio* and *Parasutterella*. Inulin also improved the integrity of the intestinal barrier, reduced inflammation, and inhibited excessive KP activation. Thus, the use of inulin as a therapeutic agent that reverses ketamine-induced damage may become a promising strategy for alleviating or treating disorders associated with cognitive deficits.

However, the bacteria’s involvement in modulating the pathway is not limited to influencing the synthesis of its metabolites. A new study on a strain of *Pigmentiphaga* sp. YJ18 has identified an unusual set of genes called the qut cluster [84]. Thanks to its presence, strain YJ18 can use QUIN as a carbon source, resulting in the ability to degrade 100 mg/L of this neurotoxic metabolite in 48 h under optimal conditions. The unique abilities of this strain may provide support in the treatment of neurodegenerative diseases.

#### 3.1.3. KYNA Precursors and Analogues

The use of precursors and analogs of KYNA represents a promising strategy for slowing the progression of neurodegenerative diseases. KYNA’s own limited ability to penetrate the BBB makes its individual use in therapy much more difficult, resulting in the development of structurally modified prodrugs and analytic derivatives that readily cross the BBB and are metabolized into neuroactive KYNA within the brain [85].

4-Chlorokynurenine (4-Cl-KYN, AV-101) is a pro-drug of 7-chlorokynurenate (7-Cl-KYNA) which is an active antagonist of the glycine site of NMDA receptors [86]. Unlike 7-Cl-KYNA, AV-101 administered peripherally, easily crosses the BBB thanks to the large neutral amino acid transporter LAT1 where it is metabolized to 7-Cl-KYNA with the involvement of KAT enzymes. Increased levels of this metabolite after treatment with the pro-drug, AV-101, were associated with neuroprotective effects in the CNS [87].

A work was initiated to optimize the efficiency of 7-Cl-KYNA penetration into the brain by combining it [88] with D-glucose and D-galactose esters as vectors for transporting the drug across the BBB [88]. The study showed that the use of this solution significantly improved the bioavailability of 7-Cl-KYNA in intraperitoneal experiments and demonstrated a protective effect and prevention of NMDA injection-induced seizures in test animals as a result of increased concentrations of 7-Cl-KYNA and KYNA in the brain.

Other studies have shown a beneficial effect of FK506 (tacrolimus), currently used as an immunosuppressive drug, whose action leads to a significant increase in neuroprotective KYNA in the brain and counteracts the inhibition of the production of this metabolite induced by 1-methyl-4-phenylpyridine and 3-nitropropionic acid [89]. This suggests the high utility of this compound in the study of alternative pathways for increasing the synthesis of neuroprotective products of the KP.

Therapeutic approaches using KYNA analogs and precursors may carry many benefits in the treatment of neurodegenerative diseases such as Parkinson’s disease [90].

Over the years, numerous novel compounds have been synthesized as analogs or precursors of KYN. Significantly less described and studied analogues in the area of metabolic pathway modulation and neurodegenerative diseases include: SZR104, which is an analog characterized by a very high ability to diffuse across the BBB [85], SZR72, which exhibits anti-inflammatory effects [91], and KYN-1, whose neuroprotective effects have been reported in tauopathies [92].

#### 3.1.4. Dietary and Nutritional Interventions

In recent years, non-pharmacological methods of modulating the KP, including a diet, have received considerable attention. In some neurodegenerative disorders, a properly selected diet and its nutrients can be an important part of therapy and can also significantly affect the production of intermediate metabolites of the KP and the gut–brain axis [93]. One of the most commonly recommended types of diets for the treatment of any neurological disorders is the ketogenic diet (KD), which is characterized by a high content of fats in the meals, which, act as energy material for the muscles and brain instead of glucose [94]. By putting the body into a state of ketosis and increasing the number of ketone bodies, this diet can suppress inflammation and reduce levels of pro-inflammatory cytokines. One of the ketone bodies involved in this process is β-hydroxybutyrate, which can inhibit the secretion of pro-inflammatory cytokines (TNF-α) and at the same time stimulate the production of anti-inflammatory mediators (IL-10, IL-1RA) [95]. As a result of these processes, a key enzyme of the KP, namely IDO, whose activity is highly induced by inflammatory processes, can be inactivated.

The significant effect of KD on TRP metabolism was demonstrated in a study conducted in animal models by Heischmann et al. [96]. Application of this diet in rats resulted in significant changes in the levels of key metabolites of the pathway, such as KYN and KYNA, compared to the control diet group. Reduced levels of KYN were observed in the plasma and hippocampus of the studied rats, accompanied by a reduction in XA concentrations. The changes observed in TRP metabolism as a result of the ketogenic diet may also be caused by calorie restriction and reduction in the availability of certain coenzymes, such as the active form of vitamin B6.

However, there are some risks of an excessively high-fat diet (HFD) leading to obesity. When HFD was administered in mice during pregnancy, it led to activation of the maternal immune system (MIA) and an inflammatory response involving the gut microbiota, resulting in increased production of pro-inflammatory proteins [97]. Activation of the MIA by interfering with the maternal TRP metabolism pathway leads to the accumulation of harmful metabolites in the fetal brain. A decrease in TRP levels and an increase in KYN levels have been observed in offspring exposed to a prenatal HFD diet, which may be due to immune activation of IDO1, as well as large increases in 3-HK, QUIN, and 3-HAA levels. Male offspring also showed abnormalities in behavioral tests, while overproduction of pathway metabolites resulted in neuronal migration disorders. Administration of 1-MT, which is an IDO1 inhibitor, to mice on an HFD diet blocked KP activity, resulting in the elimination of behavioral abnormalities.

Compared to very restrictive dietary ideas such as ketogenic diet, the Mediterranean diet represents a more balanced and holistic pattern based on vegetables and fruits, nuts and whole grans [98]. Additionally, it includes moderate consumption of fish, poultry, red wine and low intake of red and processed meats. Numerous studies indicate that due to its anti-inflammatory properties, it is associated with improved cognitive performance, particularly in episodic memory, as well as a reduced risk of cognitive decline, impairment, and neurodegenerative diseases. Evidence also suggests that adherence to the Mediterranean diet may directly influence kynurenine pathway activity. According to Sherly X Li et al., higher compliance to the Mediterranean diet, as reflected by the Mediterranean Diet Score, has been associated with lower levels of kynurenines (KYN, KYNA, AA, 3-HK, 3-HAA and QUIN), nicotinamide, and CRP [99]. An extension of Mediterraean diet, called Dutch Mediterranean Dietary Approaches to Stop Hypertension (DASH) Intervention for Neurodegenerative Delay (MIND-NL), was also studied according to kynurenine levels. It was found that greater adherence to this diet may decrease activation of the whole kynurenine pathway which is consistent with the previous findings [100].

As part of the Mediterranean diet, which includes moderate consumption of red wine, the effect of resveratrol (3,4′,5-trihydroxystilbene), a natural polyphenol with antioxidant properties found in many dietary components was also investigated [101]. It was proven that its consumption increases IDO activity, resulting in a decrease in TRP levels.

With increased dietary supply of TRP (for instance by supplementation), which is the precursor of all KP metabolites, the course and activity of this pathway can also be affected [102]. In animal models, its increased intake has been shown to increase neuroprotective levels of KYNA in the brain as a result of a dose-dependent increase in the concentration of KYN in the peripheral circulation. The mechanism of action of this strategy is based on the increased conversion of TRP to KYN caused by its increased supply. In contrast, the rapid influx of produced KYN, which is much more intensively captured by astrocytes via LAT1 transporters, accelerates the conversion of KYN to the neuroprotective acid KYNA by significantly increasing its amount.

Moreover, not only TRP, but also other amino acids (leucine, isoleucine, phenylalanine, methionine, tyrosine, alanine, cysteine, glutamine, glutamate, and aspartate), depending on the dose, can modulate the pathway as a result of blocking KYN transport and reducing KYNA concentration in the brain, as observed in animal studies [103]. Eight of these amino acids reduced the amount of KYNA in extracellular spaces by 40–60%. Of particular importance in this process were neutral amino acids of high molecular weight (leucine, isoleucine, valine, or phenylalanine), which are substrates for LAT1 transporters and compete for a binding site with TRP, resulting in a reduction in its availability in the CNS. In vitro and animal studies support the fact that supplementation of certain amino acids, resulting in blocking of KYN transport and consequently reduced KYNA production, may be a promising therapeutic strategy. This knowledge may be particularly useful in conditions that are associated with excess KYNA, which promote cognitive disorders and neurodegenerative processes.

Another important element of the diet that influences the course of the pathway is supplementation with polyunsaturated fatty acids (PUFA) [104]. The administration of fish oil (FO), rich in omega-3 and omega-6 fatty acids, resulted in a reduction in the production of TRP and KYN excessively induced by lipopolysaccharide, which led to a decrease in the TRP/KYN ratio. Fish oil supplementation led to the activation of the neuroprotective branch of the pathway, including an increase in the production of its main component, KYNA, while blocking the neurotoxic branch by reducing the production of QUIN and 3-HK in the studied animals. The study demonstrated the importance of fish oil and its components as a dietary component that strongly inhibits IDO and KMO enzymes and induces KAT. The modulating properties of fatty acids contained in fish oil may play an important role in complementary or supportive therapy for neurodegenerative diseases in the elderly.

#### 3.1.5. Physical Activity

As is well known, physical activity supports brain function and has shown therapeutic effects in many neurodegenerative diseases, such as Parkinson’s disease, Alzheimer’s disease, and multiple sclerosis, and carries many benefits for patients suffering from depression [105,106,107].

In the context of research on the modulation of the KP, there have also been numerous reports of the significant impact of regular physical activity, especially regular endurance exercise [108,109]. They affect the KP by activating the coactivator PGC-1α1 in skeletal muscles, which stimulates genes encoding kynurenine aminotransferases, as has been proven in both animal and human studies. This process results in increased expression of kynurenine transaminases (KAT I–IV), which shift tryptophan metabolism towards neuroprotective metabolites by converting neurotoxic kynurenine into KYNA and also limiting the synthesis of harmful QUIN, which has a beneficial effect on the CNS.

It was observed that high-intensity physical exercise (aerobic exercise) causes a significant increase in KYNA levels by 63% compared to QUIN, whose concentration increased by only 19% [110]. These changes lead to a significant reduction in the QUIN/KYNA ratio, which plays an important role in mental health. In addition, it was shown that in people who exercised regularly, adaptation resulted in increased expression of both KAT genes and KAT proteins in skeletal muscles compared to non-exercisers.

Other studies have analyzed the impact of physical activity in people with normal glucose metabolism and in people with type 2 diabetes [111]. It was observed that training also had a positive effect on people with diabetes, causing an increase in KYNA levels and a decrease in KYN in plasma, which suggests the high versatility of this mechanism.

Physical exercise affects not only the KAT enzyme but also IDO expression. Therefore, the effect of both acute and chronic physical exercise on changes in the levels of metabolites in the KP that depend on this enzyme was analysed [112]. A single session of very intense exercise, which induces a temporary, exercise-dependent inflammatory state, leads to the activation of IDO. This increased expression promotes the conversion of TRP to KYN, which can be metabolised to KYNA thanks to the exercise-induced activation of PGC1α in muscles. Whereas chronic exercise increases the body’s tolerance to inflammation and stimulates the production of anti-inflammatory cytokines, which may reduce IDO activity.

Other studies have shown that physical exercise decreases plasma levels of TRP and KYN and increases cerebrospinal fluid levels of metabolites such as KYNA, 3-HK, and picolinic acid (PIC) [113]. An increase in the PIC/QUIN ratio has also been observed after intense exercise and may be important in exercise’s neuroprotective role.

However, it is important to note that both a single, short workout and prolonged training will result in an increase in the KYN/KYNA ratio and activation of muscle KAT, protecting against the neurotoxic effects of KYN in the CNS [114,115].

Scientific reports about the effect of 3-HAA as a factor promoting healthy aging and slowing down the process provided the basis for investigating the relationship of how physical activity affects the production of this metabolite [116]. In *Caenorhabditis elegans* model, blocking of the gene responsible for the activity of the enzyme that degrades 3-hydroxyanthranilic acid (3-HAA) resulted in an increase in lifespan of about 30%. The same effects were observed after 3-HAA supplementation in mice. Chronic endurance training tested in two forms of CON and INC differing in exercise intensity resulted in an increase in 3-HAA levels of 85% and 134%, respectively, relative to baseline. The results of this study may be of particular relevance to middle-aged patients, whose 3-HAA levels are significantly lower.

#### 3.1.6. Stress and Immune Response

Psychosocial stress and immune factors are important in the activation of the KP [117]. Chronic stress can increase the activity of TDO and IDO enzymes, resulting in a significant increase in the conversion of TRP to KYN. In studies conducted in animal models, it was observed that chronic psychosocial stress caused an increase in TNF-α and IFN-γ, which could also be a direct cause of the increase in KYN, 3-HK, and KYNA in the blood of test animals through overstimulation of the IDO enzyme. Blocking this enzyme resulted in the reversal of these effects.

Pro-inflammatory cytokines such as IFN-γ are potent inducers of the IDO enzyme, leading to increased conversion of TRP to KYN [118]. The effects of this mechanism can be observed in patients undergoing IFN-α therapy, who exhibit reduced TRP levels, increased KYN levels, and KYN/TRP ratio, resulting in depressive symptoms.

A study by Luo et al. showed a correlation between exposure to chronic unpredictable mild stress and KP activation, which results in a shift in balance toward neurotoxic metabolites [119]. This type of stress simultaneously activates the inflammatory response, decreases KYNA levels, and promotes a significant accumulation of 3-HK and QUIN.

Similar adverse effects were obtained in children exposed to psychological stress [120]. Higher concentrations of 3-HK and 3-HAA were observed, as well as a dramatic 47-fold increase in the 3-HAA/anthranilic acid (AA) ratio, indicating a shift of the KP to its neurotoxic side. High levels of 3-HAA, with a concomitant decrease in AA, indicate excessive activation of the KMO under stressful conditions.

Concluding, the research leading to an understanding of the relationship between stress exposure, immune response and activation of the KP may be important in the search for new strategies to modulate it [121]. In contrast, stress-reducing methods such as psychotherapy or meditation as well as the use of adaptogens may not directly regulate the KP offering potential therapeutic benefit.

### 3.2. Inhibitors and Agonists of the Pathway

Three hemoprotein enzymes regulate the course of the first step of TRP catabolism, responsible for limiting the rate of this process by breaking the bond in the indole ring of TRP and introducing two oxygen groups, resulting in the formation of an impermanent N-formyl-kynurenine that is very rapidly converted to KYN [122,123].

These include TDO and IDO 1 or 2, which play different roles in TRP metabolism in the KP, and also differ in their mechanism of action and certain structural features [124]. IDO 1 is one of the key enzymes involved in the KP; it is expressed in most tissues of the body and contributes to a local decrease in TRP concentration. It is a monomeric enzyme with a very broad substrate specificity that includes both D- and L-TRP, 5-hydroxyTRP and tryptamine and its expression is mainly induced by IFN-γ, TNF-α and other pro-inflammatory cytokines.

IDO1 exists in two forms: the enzymatically active holo-IDO1, bound to a heme group, and the inactive apo-IDO1, which lacks the heme cofactor [125].

In contrast, the more recently discovered enzyme IDO2 exhibits lower substrate-affinity and significantly lower catalytic efficiency compared to IDO1. It is less induced by cytokines and its expression is not limited to a more limited range of tissues [126]. However, its precise role in the context of neurodegenerative disorders remains insufficiently understood.

TDO is a homotetrameric enzyme that, like IDO, catalyzes TRP oxidation, but in the human body its highest activity is found mainly in liver cells [127]. It is highly specific only for L-TRP, unlike the enzyme IDO1, which can metabolize both L- and D-TRP. TDO is mainly subject to hormonal regulation by glucocorticoids, and is also dependent on the concentration of TRP in the consumed meals.

The first well-known TDO inhibitor, described by Salter et al. in 1995, was a synthetic compound-fluoroindole 680C91 ((*E*)-6-fluoro-3-[2-(3-pyridyl)vinyl]-1 *H* -indole), which has high selectivity due to its lack of activity against the IDO enzyme and monoamine oxidases A and B (MAO-A/B) and also against serotonin 5-HT receptors [128]. The mechanism of action of 680C91 involves binding the catalytic site of the TDO enzyme and competing with its natural substrate L-TRP, resulting in blocking the ability to convert TRP to N-formylkynurenine. Studies in animal models have shown that oral supplementation of this synthetic compound effectively inhibited the degradation of TRP by TDO, with the parallel compound showing no activity against the IDO enzyme, demonstrating the high specificity of this compound and its suitability for further research.

In other studies evaluating the effects of 680C91, it was observed that the immediate aftermath of the inhibitor’s administration was a dose-dependent increase in TRP levels exceeding 260% of control values and a 170% increase for serotonin (5-HT) in cerebrospinal fluid [127]. In addition, there was an increase in the concentration of one of the 5-hydroxytryptamine metabolites: 5-hydroxyindoleacetic acid (5-HIAA) in the brain, confirming the maintenance of 5-HT axis activity despite the inhibition of TRP degradation by TDO. Moreover, it was observed that supplementation with high doses of TRP caused only a short-term increase in its levels in the brain, while the use of the 680C91 inhibitor provided a long-term effect of increased levels of both serotonin and TRP, which may suggest, among other things, the high antidepressant efficacy of this inhibitor. Due to the lack of interaction of this inhibitor with other tagets (MAO or 5-HT receptors), the risk of adverse interactions and side effects is minimized to a small extent.

A second inhibitor, less commonly used to study mechanisms of KP inhibition, is LM10: a small-molecule indole derivative with a favorable pharmacokinetic profile [129,130]. This compound has the ability to inhibit both TDO and IDO1, unlike other selective inhibitors that target IDO1 alone. LM10’s mechanism of action, like that of 680C91, relies on the competitive binding to the enzyme’s active site and blocking access of TRP preventing its conversion to N-formylkynurenine. Despite its many advantages, LM10 is a poorly characterized compound compared to other, better-known inhibitors. Further research of its biological activity and safety is needed, and studies should be conducted to fill in the missing clinical data and confirm the efficacy of LM10 in various disease models. However, because of its non-selective action, it may find application in situations requiring simultaneous inhibition of both IDO and TDO activity. It may also provide a basis for designing more selective inhibitors with a much better pharmacological safety profile.

In addition to indole derivatives, imidazole compounds exhibiting selectivity against TDO have also been described in the literature [131]. One promising group is imidazoindoles. Their chemical modifications have resulted in compounds that are up to 20 times more selective for TDO. In the search for effective TDO inhibitors, research teams have replaced the classic indole scaffold with its bioisostere, indazole, and developed a series of compounds based on the indazole-4-amine structure, obtaining compounds that selectively inhibit TDO. The last group of compounds are quinones, which have long been known for their ability to inhibit TDO, including compounds such as epinephrine, catechol, and L-DOPA. Contemporary research confirms the effectiveness of these inhibitors and allows for the identification of further derivatives.

Also, IDO1 and IDO2 inhibitors, by blocking the conversion of TRP to neurotoxic metabolites, have become the focus of research to evaluate their potential in the treatment of neurodegenerative diseases [132,133]. Although IDO1 is closely related to the IDO2 enzyme, with differences in key features, albeit regarding the structure of the active site, not every inhibitor will block both enzymes.

Although IDO inhibition was initially the subject of research focused only on its use in cancer therapy, as knowledge of the relationship between the KP and the development of neurodegenerative diseases has advanced, they have begun to be considered for potential therapy for Alzheimer’s disease, among others [134].

One of the precursor IDO inhibitors is the small-molecule and competitive-acting compound 1-MT [126,135]. It exists in two forms: L-1-methyl-TRP (indoximod) and D-1-methyl-TRP, which differ in their selectivity of action and pharmacokinetic profile. Studies conducted in 2007 confirmed that these 2 isomers show different specificity of action against IDO1 and IDO2 enzymes. Biochemical analyses showed that IDO2 is less sensitive to L-1-methylTRP than IDO1, while D-1-methyl-TRP reduces IDO2 enzyme activity more effectively than IDO1, making it the first selective IDO2 inhibitor of clinical interest.

In addition, studies using a mouse model of Parkinson’s disease showed that administration of 1-MT reduces the synthesis of neurotoxic QUIN in the striatum of the test animals [136]. As a result, there was a reduction in inflammatory processes and oxidative stress in cells, which minimized oxidative damage to neurons. Increased levels of TRP and serotonin due to their blocked degradation after treatment with the 1-MT inhibitor induced the synthesis of serotonergic neurotransmitters, in addition, the level of neurotrophic brain-derived neurotrophic factor (BDNF) increased, which translated into better results in the behavioral tests carried out in animals treated with this compound.

Indoximod is well tolerated, as confirmed by the results of phase I clinical trials [137,138]. Another selective holo-IDO1 inhibitor, epacadostat (INCB024360), was also developed primarily for oncology therapy, although its mechanism of action may indicate potential use in modulating the KP [132,133]. This small-molecule, orally administered classical inhibitor has a high selectivity of action, possessing a more than 1000-fold affinity advantage for IDO1 over IDO2.

Based on the known chemical structure of epacadostat, new selective IDO inhibitors are being sought [139]. One of the new scaffolds used for these purposes is N-hydroxybenzofuran-5-carboximidamide, some derivatives of which show properties of blocking the IDO enzyme.

Another potent compound that is an irreversible IDO1 inhibitor is BMS-986205, which has a significantly better pharmacokinetic profile than epacadostat and, like epacadostat, is well tolerated in phase I studies in cancer patients [140]. PF-06840003 is another promising compound that has so far been developed in clinical trials mainly for cancer treatment.

Nevertheless, attempts are being made all the time to find more selective and more effective IDO inhibitors based on already known compounds [141,142]. The use of spirophous piperidines and cyclohexanes or spirocyclic oxalamides and many other compounds provide us with new effective compounds to inhibit IDO activity.

A new class of compounds—diaryl hydroxylamines—has also been developed, in which pan-inhibitors acting on IDO1, IDO2 and TDO, as well as dual inhibitors of IDO1 and TDO, have been successfully identified [143]. The development of dual and pan-inhibitors of the enzymes of the pathway may be a promising research direction for us in KP modulation.

Another group of compounds used as a basis for developing new IDO1 inhibitors are indoles [131]. In one study, it was shown that 2-(5-imidazolyl)indole derivatives have IDO inhibitory potential. Importantly, these compounds were able to cross the BBB [144].

In contrast, another group developed a series of succinimide derivatives. The most promising compound in this group achieved IC_50_ values of 120 nM and reduced KYN levels in mice by as much as 68% in in vivo models [145]. Other compounds, on which the synthesis of new inhibitors is based, are imidazoles, including 4-phenylimidazole, identified as an IDO inhibitor as early as 1989. Interest in this molecule led to the further development of its derivatives and the creation of improved analogues. Another large group of imidazoles substituted in positions 1,5 and 4,5 was developed, of which the 1,5-substituted derivatives proved to be mostly inactive. Other base compounds used to develop inhibitors include N-hydroxyamidines, phenylbenzenesulfonylhydrazides, condensed thiazoles and aminonitriles, and iminonitriles [131].

Some tryptoline derivatives are significantly more effective in inhibiting IDO1 activity than the well-known inhibitor 1-MT [146].

However, the number of IDO2 inhibitors remains very limited, mainly due to the difficulty in obtaining the active enzyme [131]. L-1-MT inhibits IDO2 at fairly high Ki values (300–425 μM). In screening studies of compounds approved by the Food and Drug Administration (FDA), several compounds that inhibit IDO2 activity have been identified, such as tenatoprazole.

Given the high efficiency of modulation of the pathway by the use of IDO and TDO inhibitors, a search has been launched for compounds showing dual inhibition ability, both of IDO and TDO. Groups of compounds showing such properties include 1-thienyl-β-carboline derivatives [147], 1-aryl-1-H- naphtho [2,3-d][1,2,3]triazolo-4,9-dione, from which one of the most potent inhibitors has emerged [148], indole-2-carboxylic acid derivatives [149], and isoquinoline [150].

In addition to chemical synthesis, the nature is the source of dual inhibitors [151]. Analysis of the rhizomes of *Sinomenium acutum* led to the identification of an alkaloid contained therein—a natural compound that exhibited the ability to doubly inhibit enzymes.

From the roots of *Dactylicapnos scandens*, among 35 isoquinoline alkaloids, it was possible to identify an aporphine-type alkaloid that strongly inhibits IDO and TDO [152].

The marine alkaloids tsitsikammamine and wakayin also proved to be excellent dual inhibitors [153]. Modification of tanshinone IIA, which is a natural component of herbal preparations from *Salvia miltiorrhiza*, also provided a new compound with favorable properties and IDO/TDO inhibition ability [154]. In the subsequent steps of the KP, KYN serves as a substrate for three enzymes: kynurenine aminotransferase, which converts it to KYNA; KYNU, which breaks it down to anthranilic acid, and KMO oxidase, which leads to the formation of 3-HK [56].

Inhibition of all three enzymes is one strategy for modulating KP in the treatment of neurodegenerative diseases. KMO is an enzyme belonging to the oxidoreductase group, containing FAD [155].

It is involved in the conversion of KYN to neurotoxic metabolites such as 3-HK [156,157]. This is simultaneously associated with a decrease in the production of the neuroprotective KYNA. KMO inhibition has thus become an attractive therapeutic target for the treatment of neurodegenerative diseases due to its key role in regulating the balance between neurotoxic and neuroprotective metabolites [157].

The structure of most KMO inhibitors designed before the study of the exact structure of this enzyme was based on the known spatial configuration of the substrate of KMO, namely L-kynurenine [56,158]. Nevertheless, the subsequent comprehension of the crystal architecture of this enzyme in *Saccharomyces cerevisiae* (ScKMO) has opened up new possibilities for structurally targeted design of inhibitors of this oxidase. 

One of the most well-known KMO inhibitors is Ro 61-8048 (3,4-dimethoxy-N-(4-(3-nitrophenyl)thiazol-2-yl)benzenesulfonamide), a compound belonging to the sulfonamide group with very potent and selective activity. Its use is associated with inhibition of the conversion of L-kynurenine to 3-HK, which shifts the balance towards the formation of neuroprotective KYNA [159]. The study demonstrated that regular administration of Ro 61-8048, which is a selective KMO inhibitor, resulted in the accumulation of KYNA and a concomitant reduction in the neurotoxic metabolites 3-hydroxykynurenine and QUIN within the rat spinal cord of test animals.

This has led to consideration of the effectiveness of this inhibitor in treating multiple sclerosis, among other conditions [160]. There are also a number of studies reporting the effectiveness of Ro-61-8048, which by increasing kynurenine levels reduced ethanol consumption and nicotine intake, among other things, in an animal model of addiction involving mice [161,162].

And in rats orally administered Ro 61-8048 at a dose of 42 mg/kg, there was a sevenfold increase in KYNA levels in the hippocampus [57].

Studies have shown that KP inhibitors Ro-61-8048 and o-methoxybenzoyloalanine can reduce NAD+ levels in cells, resulting in induced inflammation and increased apoptosis of the hippocampus; therefore, they indicate these inhibitors also act in neuroinflammation and neuroinfectious diseases [163,164]. Thus, further research should focus on developing inhibitors and analogs targeting KP enzymes and metabolites.

However, this compound is only an experimental drug due to disqualifying features such as impenetrability across the BBB, limited bioavailability in the CNS, and too short biological half-life, resulting in the need for frequent injections in animal studies. Despite its drawbacks, it has become an excellent precursor for the inhibitor JM6, which is an oral pro-drug of Ro-61-8048. A series of studies were conducted in order to assess the effect of both single and chronic administration of JM6 to a group of test animals [165,166]. A single administration of this inhibitor at a dose of 100 mg/kg per os 5 h after application, caused an increase in KYNA concentration in the brain to 180% of the baseline value and in serum to 344%. In a subsequent experiment, JM6 was administered for 7 days at a dose of 100 mg/kg and, as in the first study, an increase in KYNA levels was observed, accompanied by a decrease in glutamate concentration. To verify the involvement of the KATII enzyme in this mechanism, rats treated with JM6 were also given kynurenine aminotransferase inhibitor, ESBA. The complete abolition of the increase in extracellular KYNA in the brain indicates that the increase in this metabolite observed in previous studies originates from de novo production in the CNS, dependent on the action of the KATII enzyme. Although neither JM6 nor its active form Ro 61-8048 penetrated the brain, their effects prevented loss of synapses and behavioral disorders, and blocking KMO increased brain concentrations of the neuroprotective KYNA and reduced glutamate release.

Sulfonamide compounds have gained considerable popularity as matrices for the synthesis of new KMO inhibitors. Among others, we identified pyridazinylsulfonamide, whose modifications translated into enhanced activity against KMO and more efficient penetration across the BBB, and the compound N-(6-(5-fluoro-2-(piperidin-1-yl)phenyl)pyridazin-3-yl)-1-(tetrahydro-2H-pyran-4-yl)methanesulfonamide, which may be considered a promising candidate for further research in Huntington’s disease therapy [167,168].

The research progress and the creation of a large database of KMO inhibitors has enabled the synthesis of new and improved substances [169]. A pharmacophore-based UPF648 inhibitors allowed the selection of two compounds that are hippuric analogues. 3,4-dichlorohippuric acid blocked KMO activity with moderate potency (Ki = 34 μM), while 3,4-dimethoxyhippuric acid showed significantly weaker inhibitory activity (Ki = 1 mM). The synthesis of new compounds with KMO inhibitory activity based on the pharmacophores of already known inhibitors may be beneficial in the form of new drugs for clinical trials on their potential therapeutic use.

However, not only synthetic chemical compounds can become KMO inhibitors. A study by Puopolo et al. evaluated the effect of cannflavin A isolated from fiber hemp as a compound that inhibits the KMO enzyme [170]. It proved to be a good inhibitor comparable to Ro 61-8048. The results suggest that cannflavin A may become a natural KMO blocker, opening up new possibilities in the treatment of neurodegenerative diseases associated with dysfunction of the KP.

Rebai et al. verified using in silico and in vitro methods to test whether flavonoids, which are natural compounds belonging to the polyphenol group, can also act as KMO inhibitors [171]. Studies including docking and molecular dynamics identified several compounds that effectively competitively inhibit KMO. 3′-hydroxy-α-naphthol and 3′-hydroxy-ss-naphthol showed the most effective activity against KMO, suggesting that such compounds may form the basis for further research into the treatment of neurodegenerative diseases involving excessive KMO activation.

Another widely described inhibitor is 2-(3,4-dichlorobenzoyl)-cyclopropane-1-carboxylic acid known as UPF648. Its action is to block the binding of L-KYN by interacting strongly with the FAD cofactor and thereby effectively inhibiting the catalytic activity of KMO [158,171]. In vivo studies in animal models have shown that UPF648 administration leads to a shift in the KP toward neuroprotection—by reducing the levels of neurotoxic 3-HK and its subsequent metabolite QUIN while significantly increasing KYNA levels in the brain and liver of rats. In addition, studies on transgenic fruit flies with induced Huntington’s disease symptoms showed that UPF648 reduces the intensity of disease symptoms and increases neuronal survival, thereby suggesting that KMO inhibition may become an effective pathway modulation strategy for treating neurodegeneration. Although UPF648 demonstrates favorable pharmacokinetic properties, its therapeutic application remains limited due to non-selectivity toward other FAD-dependent enzymes and potential adverse effects. Nonetheless, studies with this inhibitor strongly support the concept that KMO inhibition is one of the most promising strategies for the treatment of neurodegenerative disorders. Iantellamide A is a natural and selective KMO inhibitor, structurally related to octopamine, a neurotransmitter. It was isolated from the Australian sea sponge *Ianthella quadrangulata* and evaluated for its effects on KP enzymes [172]. Its unique biological activity arises from the presence of two bromine atoms, a sulfate group, and an octopamine moiety within its structure. Iantellamide A showed effective and potent activity against KMO characterized by an IC value of 1.5 μM. In rats, administration of Iantellamide A significantly increased endogenous KYNA levels in the brain, supporting its potential as a neuroprotective agent in neurodegenerative diseases. Recently, there has been a trend in the discovery and synthesis of new KMO inhibitors with a simplified structure compared to older compounds [166,172]. Most of these molecules contain a carboxylic acid group, which restricts their ability to cross the BBB. To address this limitation, researchers employ computational approaches, including in silico screening with the Zinc15 database. These strategies have led to the identification of compounds such as ZINC-71915355 and ZINC-19827377, which show high KMO affinity without generating toxic by-products like hydrogen peroxide. ZINC-71915355 has been identified as a molecule that potentially crosses the BBB.

Another enzyme of therapeutic interest is kynurenine aminotransferase (KAT), which catalyzes the conversion of L-KYN to KYNA. KYNA exerts neuroprotective effects by antagonizing NMDA and α7-nicotinic receptors. In this group of pyridoxal phosphate (PLP)-dependent enzymes, there are 4 isoforms: KAT I, KAT II, KAT III and KAT IV [173].

Due to its predominant expression in the CNS and distinct catalytic features, KAT II has become the most extensively studied isoform in this enzyme family [174,175,176,177]. Inhibition of the activity of this enzyme leads to a decrease in the level of KYNA, which, despite its neuroprotective properties, when present in excess, especially in the hippocampus or striatum, can contribute to glutamatergic and cholinergic transmission disorders. Although KYNA has protective properties, its excessive accumulation—as observed in certain neurodegenerative diseases and schizophrenia—contributes to cognitive dysfunction. This effect arises from antagonism of NMDA and α7 nAChR receptors, leading to hypoglutamatergia and reduced dopamine levels. This condition promotes an increase in psychotic symptoms in these disorders.

The importance of KATII inhibition has made it a potential therapeutic target for the treatment of cognitive disorders and schizophrenia [177]. The first studies on pharmacological modulation of the KP by altering KYNA levels, in effect inhibiting KAT enzyme activity, were based on non-selective PLP-dependent aminotransferase inhibitors. One of the most common compounds used in these studies was aminooxyacetic acid. It is characterized by its ability to cause irreversible blockade of a broad spectrum of pyridoxal phosphate-dependent aminotransferases, including all four isoforms of kynurenine aminotransferase.

Regardless of the administration route (intracerebral or peripheral), AOAA inhibited KAT activity and blocked the conversion of L-KYN into KYNA, significantly lowering its concentration in brain structures [178,179,180]. Despite the effective mechanism of action, the use of AOAA carried a number of side effects such as damage to nerve cells in the striatum and disturbances in the balance of neurotransmitters. This resulted in the induction of seizures in experimental animals. Due to the occurrence of neurotoxicity and lack of selectivity, the use of acid is limited only to experimental studies of the KP and is not being considered as a potential candidate for the treatment of neurodegenerative disorders. Despite the existence of many limitations, studies involving it have provided researchers with valuable clues for further inhibitor development and pointed the way to the design of new compounds with a better safety profile and selectivity of action, which later moved to the development of much more efficient compounds such as: (S)-ESBA, BFF-122 or PF-04859989. A major breakthrough occurred in 2006 with the development of the first selective, reversible KAT II inhibitor, (S)-4-(ethylsulfonyl)benzoylalanine ((S)-ESBA), which exhibited an IC_50_ of 6.1 μM against rat KAT II [181].

Administration of this compound to the test rats led to an effective 35% reduction in KYNA levels, which secondarily induced a 270% increase in extracellular dopamine levels in the test animals’ striatum. This may have resulted from the unblocking of NMDA receptors and regulation of glutamatergic transmission [181,182,183,184]. Despite good selectivity and inhibition efficiency in animal studies, the compound (S)-ESBA did not show a similar effect on its human counterpart KATII (hKATII) potentially due to differences in the structure of the enzyme’s active site across species.

Similar effects on KP were demonstrated by the small molecule inhibitor BFF-816, which reduced extracellular KYNA levels by approximately 30% in the brains of rats after administration of 30 and 50 mg/kg of the compound [185]. In addition, its potential was confirmed by behavioral tests which showed that daily administration of BFF-816 for several days resulted in a reduction in the escape time from the Morris water maze, which meant an improvement in learning ability and cognitive memory in the tested rats. Such pro-cognitive effects of this inhibitor may be of great importance in the treatment of diseases involving cognitive deficits, such as Alzheimer’s disease. The IC_50_ value for this compound was 13.4 μM, which indicates its moderately strong effect compared to other inhibitors.

The next generation of KATII inhibitors included the fluoroquinolone compound BFF-122, which proved to be almost six times more potent than previous analogues such as BFF-816, with an IC_50_ value of only 1 μM [186]. In addition, it was shown that this compound is a good inhibitor of human recombinant hKATII (IC_50_ = 0.91 μM), making it more useful in research on the therapeutic use of KAT II inhibitors in the treatment of neurodegenerative diseases. A distinguishing feature of this inhibitor, apart from its greater potency, is its good solubility and metabolic stability.

The last compound on which most scientific research is focused is (3 S) -3-amino -1-hydroxy-3,4-dihydroquinolin-2(1 *H*)-one (PF-04859989), which is an irreversible, potent, and selective inhibitor of KAT II with the ability to diffuse across the BBB [187]. Its mechanism of action is based on covalent binding to the cofactor of kynurenine aminotransferase at its active site, resulting in irreversible inhibition of this enzyme. Preclinical studies conducted by Dounay et al. showed that it reduces KYNA concentrations in the brain by approximately 50% at a dose of 10 mg/kg and normalizes ketamine-induced cognitive deficits. PF-04859989 is also characterized by a long half-life in the CNS and minimal side effects in standard tests. Due to its high efficacy in reducing KYNA levels, PF-04859989 may also be useful in the treatment of cognitive disorders associated with KYNA excess, but further studies are needed to confirm its safety.

Due to their excellent safety profiles, new inhibitors are being sought among compounds already known on the market [188]. One of these is N-acetylcysteine, which is widely available and has known pharmacological properties, and has been studied for its effects on the KAT II enzyme due to its beneficial effects in the CNS by regulating glutamate, among other things. Its use effectively inhibited KAT II activity both in recombinant human KAT form and in human and rat brain homogenates.

All generations of KAT II inhibitors have evolved significantly from reversible but interspecies-limited (S)-ESBA, through more stable compounds that are effective not only against rat but also human KAT (BFF-122), to very potent and irreversible inhibitors (PF-04859989) with excellent pharmacokinetic properties in the CNS [189,190]. Studies conducted with each of these compounds have contributed to a better understanding of the role of KYNA in neurodegenerative disorders and cognitive impairment and provide a starting point for further clinical research into the development of therapies for neurological diseases. The last enzyme, whose inhibition shifts TRP metabolism toward the production of the neuroprotective acid KYNA while reducing the formation of neurotoxic metabolites, is KYNU, which belongs to the PLP-dependent aminotransferase family of enzymes. It catalyzes the conversion reaction of both L-kynurenine to anthranilic acid and 3-hydroxykynurenine to 3-HAA. Its inhibition reduces the synthesis of QUIN and 3-HK and increases the availability of L-KYN for the previously described group of enzymes, KAT aminotransferases.

There are two major inhibitors whose actions effectively modulate the KP, namely nicotinylalanine and meta-nitrobenzoylalanine [191]. Nicotinylalanine is an analog of KYN in which a nicotinyl group is introduced at the carboxyl residue. It has the ability to inhibit both KMO and KYNU, leading to a reduction in the formation of neurotoxic QUIN and 3-HK, while increasing substrate availability for kynurenine aminotransferase and leading to the overproduction of KYNA. In studies on rats, nicotinylalanine increased extracellular KYNA concentrations by over 50%, while also demonstrating anxiolytic and anticonvulsant effects.

Three other analogs of KYN were also synthesized, differing in the position of the nitro group on the alanine benzoyl ring: ortho-, meta- and para- nitrobenzoylalanine [192]. Each isomer had a basic KYN backbone, but differed in the position of the nitro group. This modification significantly affect the affinity of a given analogue for the enzymes of the KP. Of these, m-nitrobenzoylalanine the strongest activity, inhibiting the KMO enzyme with an IC_50_ of ~0.9 μM and at the same time inhibiting kynureninase more weakly with an IC_50_ of ~100 μM, making it an excellent KMO inhibitor and a moderate KYNU inhibitor.

Meta-nitrobenzoylalanine shows an analogous mechanism of action to nicotinylalanine by directing TRP metabolism toward the production of KYNA while decreasing QUIN concentrations [193,194,195]. Thanks to these properties, its administration in the conducted studies resulted in a significant increase in KYNA in the brain, resulting in anticonvulsant and sedative effects in the test animals.

## 4. Therapeutic Potential of Metabolic Pathway Modulation in Neurodegenerative Diseases

**Figure 2:**
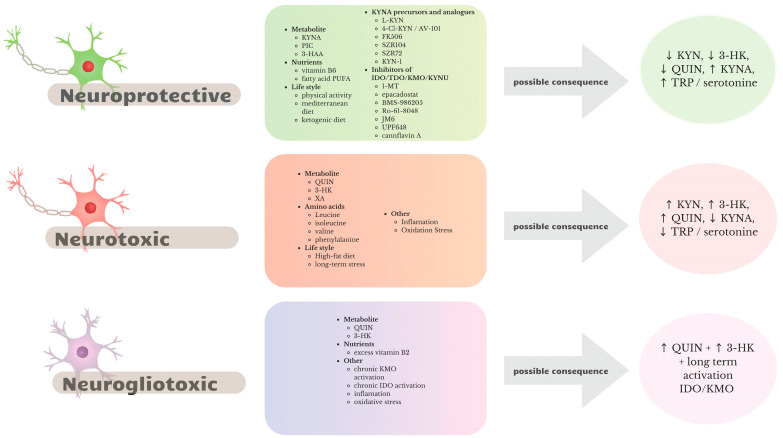
The Role of the Kynurenine Pathway in Brain Homeostasis. ↓ decrease, ↑ increase.

Neurodegenerative diseases are a group of incurable, progressive disorders of the nervous system characterized by the loss of neuronal function, leading to severe disability and ultimately death [2]. The best known disease entities include Alzheimer’s disease, Parkinson’s disease, and ALS. In addition to the aforementioned disease entities, Huntington’s disease and multiple sclerosis also fall into the category of neurodegenerative diseases [2]. There is growing support for the involvement of the KP in their pathogenesis (Figure 2).

Despite significant differences in clinical manifestations and molecular mechanisms, these diseases share a profound impact on patients’ lives and pose a major challenge to healthcare systems. A better understanding of the pathogenetic mechanisms, including the role of the KP, may open new therapeutic possibilities in the future.

### 4.1. Alzheimer’s Disease

Alzheimer’s disease is the most common disease entity with symptoms of dementia, accounting for approximately 60–70% of dementia cases worldwide [196]. Alzheimer’s disease is pathomorphologically associated with the deposition of amyloid plaques in the brain [2]. They are composed of β-amyloid peptide and neurofibrillary tangles of hyperphosphorylated tau protein. This results in neurodegeneration and loss of synaptic connections. Accompanying inflammatory processes, oxidative stress, and mitochondrial dysfunction additionally accelerate disease progression.

An imbalance between neuroprotective and neurotoxic metabolites in tryptophan metabolism is often observed in Alzheimer’s disease and is associated with accelerated cognitive decline [12]. Decreased levels of KYNA and increases in the toxic effects of QUIN and 3-hydroxykynurenine were found in most Alzheimer disease patients. In preclinical studies on mouse models of AD, the effects of pharmacological inhibition of the enzyme IDO1 activated by amyloid and tau protein aggregates in astrocytes were examined [197]. These analyses showed that inhibition of IDO1—using inhibitors originally developed for oncology—regulated kynurenine synthesis, restored astrocytic metabolic homeostasis, and significantly improved synaptic plasticity and cognitive function in test animals. In light of these results, IDO inhibitors that are in clinical trials as oncology drugs may be considered as potential therapeutic agents for neurodegenerative diseases. Coptizine, an IDO inhibitor attenuates symptoms resembling those occuring in Alzheimer Disease. Coptizine’s effect on IDO results in inhibition of microglia and astrocyte activity and amyloid β production [198].

Elevated brain TDO levels have been observed in both Alzheimer’s disease mouse models and patients, suggesting a role of KP in the formation of neurofibrillary tangles and senile plaques [165]. TDO inhibition results in reduced progression of neurodegeneration in mouse models of Alzheimer’s disease. Administration of the TDO inhibitor APP/PS1 to mice for 4 weeks counteracted hippocampal-dependent cognitive deficits.

Reducing KYNA levels through inhibition of KAT II (e.g., with BFF816) may enhance cognitive performance [165]. Studies have shown that the use of 4-chloro-HAA synthetic 3,4-dioxygenase inhibitor 3-HANA inhibits QUIN synthesis and reduces functional deficits in an experimental model of spinal cord injury in guinea pigs. Studies demonstrated that 4-chloro-3-HAA, a synthetic 3-HANA 3,4-dioxygenase inhibitor, suppresses QUIN synthesis and reduces functional deficits in a guinea pig model of spinal cord injury.

In a study by Zwilling et al. using APPtg transgenic mice harboring overexpression of human amyloid precursor protein and two familial Alzheimer disease mutations, the presence of which determines overproduction of β -amyloid oligomers and formation of amyloid plaques, investigated the effects of the KMO inhibitor 2-(3,4-dimethoxybenzenesulfonylamino)-4-(3-nitrophenyl)-5-(piperidin-1-yl)methylthiazole (JM6) as a potential therapeutic agent for the treatment of Alzheimer disease [165,199]. Animals treated with the JM6 compound showed significant improvements in spatial memory function and a reduction in anxiety behavior. JM6 also effectively inhibited the decline in synaptophysin levels in the cerebral cortex and hippocampus, thus providing protection against synaptic degeneration. Administration of 75 mg/kg JM6 for 120 days to APPtg mice resulted in an increase in KYNA levels in both brain and plasma, with no change in 3-HK and QUIN levels or KMO activity. This mechanism suggests that JM6 effectively increases neuroprotective metabolite levels without disrupting the balance with neurotoxic metabolites. Preclinical studies suggest that the use of the KMO-inhibiting prodrug JM6 may be a useful and safe therapeutic strategy in Alzheimer’s disease due to its modulation of the KP and its neuroprotective effects [199]. By causing a moderate and long-lasting increase in KYNA in the absence of disruption from metabolites such as 3-HK and QUIN, JM6 reduces the occurrence of potential side effects in the CNS.

### 4.2. Parkinson’s Disease

Parkinson’s disease is the second most common neurodegenerative disease after Alzheimer’s disease. Its characteristic symptoms include resting tremor, muscle rigidity, bradykinesia, and balance disturbances [200]. As the disease progresses, non-motor symptoms also develop, such as sleep disorders, depression, or dementia. The pathogenesis of Parkinson’s disease focuses on the degeneration of dopaminergic neurons in the gray matter of the midbrain and the presence of Lewy bodies—intracellular aggregates of the protein α-synuclein.

A number of studies in animal models of Parkinson’s disease phenotype have suggested that an increase in neuroprotective KYNA and a decrease in QUIN levels have an impact on the development and progression of the disease [201]. Analyses by Silva-Adaya et al. in a 6-hydroxydopamine (6-OHDA)-induced rat model of Parkinson’s disease evaluated how administration of the precursor KYNA (L-kynurenine) in combination with the organic acid transport inhibitor probenecid (PROB) affects the Parkinson disease model. L-KYN (75 mg/kg) and PROB (50 mg/kg) were administered for one week, with a single injection of 20 μg 6-OHDA (2 μL) on day 2. On day 14, analyses were performed to assess the effect of L-KYN + PROB on 6-OHDA-induced damage. In the group of animals treated with these compounds, a marked alleviation of rotational behavior that is an indicator of motor deficits was observed, as well as a smaller decrease in dopamine levels compared to the group of animals treated with 6-OHDA alone. After twenty-eight days immunohistochemical tests against GFAP (glial fibrillary acidic protein) and hematoxylin/eosin staining showed that the applied therapy reduced reactive gliosis, and Fluoro Jade staining showed that there was a reduction in striatal neurodegeneration. The results demonstrate that L-KYN combined with probenecid effectively reduces dopaminergic damage, most likely by increasing endogenous KYNA levels. Subsequent studies on the therapeutic application of KP modulation evaluated the effects of the indoleamine-2,3-dioxygenase-1 inhibitor, 1-methylTRP, on the condition of mice with a 6-OHDA-induced model of Parkinson’s disease [90]. Inhibition of IDO1, induced by long-term administration of 1-MT at various doses, resulted in reduction in oxidative stress, elevation of dopamine and homovanillic acid levels, and reduction in QUIN levels, among other effects. Behavioral tests showed significant improvements in activity and motor coordination in the test animals. In another study using a rotenone-induced PD model, the effects of a new TDO enzyme inhibitor, NTRC 3531-0 administered chronically for 21 days in oral form, were analyzed. By inhibiting TDO activity, this therapy significantly increased brain and plasma tryptophan levels and the number of tyrosine hydroxylase-positive (TH+) cells, suggesting enhanced neuroprotection of dopaminergic neurons. Scientists analyzed the effects of 12 weeks of vitamin B3 supplementation on the KP and inflammatory markers in Parkinson’s disease patients undergoing deep brain stimulation [202]. In the group of patients receiving vitamin B3 supplementation, there was a decrease in inflammatory markers such as TNF-α and an increase in neuroprotective KYNA. Previous studies have also demonstrated the effect of this vitamin on improving physical condition in Parkinson’s patients. These results suggest that the supply of vitamin B3 favorably affects the balance of KP metabolites, which may be important in the development of therapies based on KP modulation to support the treatment of Parkinson’s disease.

The dynamic development of pathway inhibitors has led to the creation of complex compounds that inhibit more than one enzyme [147]. Research on a group of modified 1-thienyl-β-carboline derivatives has identified a compound CZ-17, named 1-(2,5-dimethylthiophen-3-yl)-9 H -β-carboline, which inhibits IDO1 (IC50 = 0.33 μM) and TDO (IC50 = 1.78 μM) enzymes with moderate potency and reduced the KYN/TRP ratio in BV2 and HepG2 cells. CZ-17 showed neuroprotective activity in a neuronal cell model (PC12), while in a PD model in zebrafish, it corrected abnormal motor functions. The compound was evaluated for toxic effects and no harmfulness was observed at the doses used, while the permeability test confirmed the ability of CZ-17 to cross the BBB. The properties of this dual inhibitor make it a promising candidate for a drug for Parkinson’s disease acting through modulation of the KP.

Pharmacological KMO inhibition has consistently been shown to enhance KYNA production [13]. The results of the study indicate that KMO inhibitors have an effect on reducing the symptoms of muscle tone disorders in hamsters, suggesting the possibility of using KMO inhibitors to treat movement disorders associated with inoperable striatum in Parkinson’s Disease.

### 4.3. Huntington’s Disease

Huntington’s disease is a genetic disorder characterized by motor dysfunction, cognitive decline, and psychiatric disturbances [203]. HD pathology is closely related to excitotoxicity, partly caused by excessive levels of QUIN. Modulators targeting enzymes such as KMO are being considered for their ability to compensate for the pathway, in this case, a shift toward KYNA.

Huntington’s disease has been linked to increased KMO enzyme activity, increasing neurotoxic products, similar to Alzheimer’s disease: QUIN and 3-HK [56,204]. Therefore, research is underway to evaluate the potential efficacy and neuroprotective effects of KMO inhibitors, along with other strategies showing promise in treating Huntington’s disease. The inhibitor JM6, a prodrug of Ro 61-8048, has been proposed as a potential therapeutic approach in Huntington’s disease and has been investigated in this regard using R6/2 transgenic mice, which are characterized by rapid progression of neurological symptoms and early death. The compound was administered to the animals from 4 weeks of age, orally at doses of 7.5 or 25 mg/kg. Analysis of survival curves (Kaplan–Meier) showed a significant correlation between increased lifespan and the dose of the compound. This effect was also maintained in the group of test animals that had previously undergone environmental enrichment to prolong survival in this model. As in the case of Alzheimer’s disease studies, JM6 protects against excessive loss of synaptophysin in both the striatum and cerebral cortex, as confirmed by histological studies in 12-week-old R6/2 mice treated with this compound, while the reduced number of Iba1-positive cells indicates a decrease in microglial activity [165].

Surprisingly, neither Ro 61-8048 nor its prodrug JM6 crosses the BBB, suggesting that peripheral KMO inhibition alone may provide sufficient neuroprotection [165,199]. This results in the accumulation of KYN in the blood, which is transported to the CNS and converted to KYNA there, without the need for the inhibitor to cross the barrier. One of the key studies in the field of pathway modulation and its potential significance in the treatment of Huntington’s disease was the demonstration that both pharmacological and genetic inhibition of KMO leads to a shift in metabolic balance towards neuroprotection and significantly alleviates neurodegenerative changes.

The authors of the study compared the efficacy of three KMO inhibitors: UPF-648, JM6, and Ro 61-8048. All of the compounds tested showed similar, strong neuroprotective effects, shifting the KP balance toward KYNA production [199,205]. The UPF-648 inhibitor led to nearly 90% protection against neuronal degeneration and a marked increase in KYNA production. The results suggest that inhibition of KMO and TDO enzymes and promotion of KYNA synthesis is a universal strategy for modulating the pathway, particularly promising in the treatment of Huntington’s disease.

Joining the group of inhibitors being investigated in the context of new potential therapies for Huntington’s disease is compound CHDI-340246, which is a novel selective inhibitor of KMO [12]. Preclinical studies conducted in Huntington disease transgenic mice confirmed that oral administration of this potent inhibitor plays a key role in modulating the KP in both the central and peripheral nervous systems. Treatment with this compound resulted in decreased levels of neurotoxic KP products such as QUIN and 3-HK and also caused an increase in KYN and KYNA, acting as a neuroprotectant. Most significantly, both chronic and short-term administration of CHDI-340246 restored normal electrophysiological functions in mouse models of Huntington disease, confirming its usefulness as a potential therapeutic agent.

Despite the promising results of all the preclinical studies, so far, no decision has been made to initiate human clinical trials [165]. However, research institutions focused on the search for drugs to slow the development of Huntington disease are emphasizing the importance and potential of KMO as a therapeutic target and aiming to develop an effective drug, as evidenced by studies on the compound CHDI-340246.

### 4.4. Multiple Sclerosis

Multiple sclerosis is considered an autoimmune demyelinating disease that also shows signs of progressive neurodegeneration, especially in later stages [206]. Studies have shown abnormalities in the KP in MS patients, with altered levels of KP metabolites in cerebrospinal fluid and plasma. And, as in multiple sclerosis, decreased KYNA levels and increased QUIN levels were observed, supporting an imbalance between neuroprotection and neurotoxicity. Immunomodulatory therapies that also include effects on KP metabolism- such as KMO inhibitors are being considered as a way to reduce inflammation and protect neurons in multiple sclerosis [207]. Clinically, it is characterized by progressive memory impairment, cognitive and behavioral disorders. In the later stages of the disease, speech paralysis, difficulty moving occur, which ultimately leads to complete dependence on third parties. In a preclinical study using the experimental mouse model of autoimmune encephalomyelitis (EAE) and another mouse model of multiple sclerosis, high hyperactivity of the KP was observed, resulting in the accumulation of neurotoxic 3-hydroxy-kynurenine and QUIN [208]. In patients with multiple sclerosis during remission, increased expression of KAT I and KAT II) and increased concentration of KYNA are also observed; however, these parameters vary depending on the phase of the disease.

Analyses showed that administration of 1-methyltryptophan (1-MT) at the appropriate disease stage-after achieving immune tolerance—alleviated symptoms and led to significant clinical improvement [207]. The observed increase in KYNA levels in brain and spinal cord after Ro 61-8048 (a KMO inhibitor) treatment in EAE mice, together with the effects of IDO inhibition by 1-MT, suggests that multiple sclerosis therapy should be based on carefully timed KP modulation. The authors of the study emphasize that the effectiveness of such therapy may consist of IDO1 inhibition at the appropriate stage to induce tolerance and also KMO inhibition at the time of overproduction and accumulation of neurotoxic 3-HK and QUIN products. For now, there is a lack of evidence from clinical trials confirming the effectiveness of the above-described strategies, but the preliminary results obtained so far encourage the development of these approaches in the treatment of not only multiple sclerosis, but also other diseases involving neurodegeneration. In response to the need to find an effective therapy to support the remyelination process in multiple sclerosis patients and show protective effects against neurodegeneration, Edaravone, a drug with potent antioxidant properties used in some countries for the treatment of ALS, was investigated [209]. The effects of this drug were studied in an animal model of cuprizone-induced demyelination. Analyzing the effect of the test drug on the KP, a significant reduction in the levels of toxic metabolites such as QUIN and AA was observed, while KYNA increased. The changes in the metabolite ratio were due to a decrease in the activity of IDO and KMO enzymes. Edaravone treatment significantly reduced cognitive deficits, improved motor skills, and enhanced myelin restoration in rats, highlighting its potential as an adjunctive therapy in multiple sclerosis.

### 4.5. Safety in Clinical Trials

One of the few KP-modulating compounds that has advanced to the clinical trial phase is compound KNS366, a selective KMO inhibitor. It successfully passed phase I trials, demonstrating an excellent safety profile, high tolerability, and most importantly, sustained KMO inhibition with a significant reduction in 3-HK. The results of these studies testify to its potent action and high efficacy, which gives hope for the development of therapies targeting modulation of the KP [210]. A second compound that has relatively recently undergone phase I clinical trials is KYN-5356, which is an inhibitor of the enzyme KAT II. In studies in healthy volunteers, the compound was shown to be safe, to penetrate well into the CNS, and to be highly tolerated and effective as evidenced by a significant decrease in KYNA in cerebrospinal fluid. Significant improvements in cognitive function were also reported, demonstrating that KAT inhibition is both achievable and effective. Although research on this compound is currently focused only on the treatment of schizophrenia and has not been evaluated in the context of neurodegenerative diseases, it may be a future direction in the treatment of those disorders accompanied by cognitive impairment [211]. Clinical trials of KP inhibitors conducted to date have focused on evaluating the safety profile and pharmacokinetics. Both the KNS366 inhibitor and the compound KYN-5356 in phase I showed a very strong biological effect and an excellent safety profile in terms of the absence of adverse events.

However, many inhibitors have limitations that prevent them from advancing to the clinical trial phase. A significant proportion exhibit limited CNS penetration and require carriers or prodrugs [212,213]. In the case of IDO inhibitors, a major limitation is the complex immune response that arises, as IDO1 blockade can suppress inflammation, but unfortunately can lead to a reduced immune response and many adverse effects associated with the phenomenon of disruption of the immune mechanism.

Modulation of the KP in neurodegenerative diseases offers many therapeutic benefits; however, its application requires caution due to the complex role of KP metabolites in neurobiology and the delicate balance between neurotoxicity and neuroprotection. Therefore, many clinical trials are needed to understand the role of multiple processes, safety and efficacy of such interventions in the TRP metabolism pathway.

## 5. Research on New KP-Related Biomarkers

Peripheral biomarkers may have an important role in selecting an individualized treatment plan for people with neurodegenerative diseases such as Alzheimer’s disease or Parkinson’s Disease [214]. However, the clinical application of biomarkers remains underemphasized, partly due to the disproportionate focus on drug development compared to clinical biomarker research. The demonstration of variability in the levels of KP metabolites indicates that they may have important roles in the pathogenesis of Alzheimer’s disease [198]. KP metabolites exhibit different properties—some are neurotoxic while others regulate oxidative stress.

KP is responsible for the catabolism of more than 90% of TRP. A useful marker of KP dysfunction may be an elevated KYN/TRP (K/T) ratio, which at the same time may represent an increased risk of CNS diseases. An elevated K/T ratio is therefore associated with an increased risk of CNS disorders. Increased conversion of TRP to KYN leads to an increase in the concentration of KYN in the brain, through its easy permeability through the BBB.

The K/T ratio is a measure of the formation of later KP metabolites [198]. An elevated K/T ratio has been highlighted as an important marker in AD research. A study by Knapskog et al. found that KYNA and PA concentrations were higher in AD patients than in controls and an increase in KYNA was associated with slower Alzheimer’s disease progression, which may suggest that an increase in Parkinson’s Disease also affects slower disease progression [215].

Schwarz et al. examined serum levels of TRP, KYNA, 3-HK, QUIN and Parkinson’s Disease in Alzheimer’s disease patients and a control group consisting of people with severe depression and subjectively impaired cognitive functions [215,216]. Levels of 3-HK were significantly elevated in Alzheimer’s disease subjects compared to the control group. The increase in 3-HK, as a metabolite of KP readily permeable to the BBB, may suggest an increase in QUIN in the brains of patients as QUIN is a downstream metabolite of KP.

A potential link has been hypothesized between PD and altered KP metabolite profiles in the blood. With age, an increase in blood IDO levels is observed in Parkinson’s Disease [217]. In fruit flies, an increased urinary KYN/KYNA ratio was found. Other studies have presented an increase in KYNA and 3-HK levels in cerebrospinal fluid, which may exacerbate oxidative processes in Parkinson’s Disease patients. Increased K/T, KYN, AA and KYNA ratios have been found in the serum of patients with Parkinson’s Diseases well as Alzheimer’s disease. recently, an increase in the QUIN/KYNA and QUIN ratio has been observed in the plasma of people with Parkinson’s Disease. The results of other studies report decreased levels of KYNA in cerebrospinal fluid with increased levels of KYN, KYNA and QUIN in the serum of Parkinson’s Disease subjects. Further research on biomarkers is needed to diagnose early PD, and continued profiling is needed to create effective pharmacological interventions.

## 6. Difficulties in Understanding the Role of KP in Neurodegenerative Diseases

Evidence from multiple studies suggests that downstream KP metabolites contribute to conditions such as Parkinson’s disease, highlighting them as potential therapeutic targets. The prospect of modulating KP to decrease QUIN levels and increase KYNA levels in the brain suggests a new solution to reduce excitotoxicity and enhance neuroprotection [217].

One of the challenges in the study of KP is the different activities of its enzymes depending on the type of cells. In astrocytes and hippocampal neurons, TDO has the greatest influence, while microglia and macrophages show a preponderance of IDO activity [13]. Difficulties in understanding the effects of KP on neurodegeneration are caused by the formation of the neuroprotective metabolites KYNA and picolinic acid, but also the neurotoxic metabolites QUIN and 3-HK [12]. Minimal structural changes within quinoline-derived metabolites can profoundly alter their biological activity and stability. It can seriously affect their biological activity and stability. Even subtle modifications, such as repositioning a hydroxyl group, can determine whether a metabolite exerts excitotoxic or neuroprotective effects [218]. Depending on their concentrations, 3-HK and 3-hydroxyanthranilic acid (3-HANA) have pro-oxidant as well as antioxidant properties. The oxidation of 3-HK produces superoxide anion and hydrogen peroxide, which have toxic effects on neurons, but 3-HK can also eliminate free radicals and inhibit lipid peroxidation. In addition to its pro- and antioxidant properties, 3-HANA also exhibits anti-inflammatory effects. The concentration of 3-HANA is reduced in the blood of post-stroke or chronic brain injury patients, while higher levels of this KP metabolite have been shown in HD patients. The dual role of 3-HK and 3-HANA may depend on the redox state of the cell [219]. Another limitation in understanding the role of KP is the lack of consistent and reproducible markers [220]. A significant difference has been observed between serum and cerebrospinal fluid concentrations of KP metabolites among patients. Their concentrations depend on age and disease severity.

Researchers also face discrepancies between experimental findings and the widely accepted KP model [221]. Studies have shown that QUIN concentrations in the brain do not fall as assumed after pharmacological inhibition or genomic elimination of KMO. Although KYNA levels can increase up to 100-fold, suggesting a shift toward the neuroprotective branch, QUIN concentrations often remain unchanged. The complete removal of genomic KMO results in a decrease in QUIN concentration by only about 20%, at the same time the concentration of KP metabolites, which have received less attention so far—anthranilic acid and picolinic acid—increases.

The development of safe and effective therapies for CNS disorders remains an urgent priority. The emergence of targeted therapies, however, requires a better understanding of the pathogenesis of these conditions [219]. Difficulties in understanding the role of KP in neurodegenerative diseases contribute to problems in developing safe and effective therapies. KP metabolites have varying abilities to penetrate the BBB. TRP, KYN, and 3-HK penetrate the BBB through the large amino acid transporter, AA crosses the BBB by passive diffusion. In contrast, KP metabolites such as 3-HANA, KYNA, and QUIN exhibit poor BBB permeability.

Intensive activation of KMO allows cells to meet temporary higher energy demands, but prolonged overactivation causes oxidative damage and accelerates apoptosis [13]. This mechanism may play a serious role in the context of neurodegenerative diseases due to the sensitivity of neurons to oxidative stress and energy depletion. The possibility of pharmacological inhibition of KMO in the treatment of neurodegeneration looks promising. Studies by Campesan et al. showed that KMO inhibition reduces Huntington disease symptoms in a *Drosophila melanogaster* model. However, not all KMO inhibitors can penetrate the BBB, so it is important to find compounds with this ability.

Interventions targeting the KP can also exert negative cellular effects. The effect of IDO is particularly related to antioxidant capacity. However, IDO-derived KYN metabolites already exhibit both antioxidant and pro-oxidant activities [13]. Excess 3-HKYN, 3-HANA and QUIN increase neuronal dysfunction and accelerate apoptosis. However, IDO inhibition can cause a decrease in neuronal and astrocyte function and viability, which is associated with reduced levels of NAD+, which is synthesized de novo by IDO. These adverse effects were more pronounced in neurons than in astrocytes, suggesting that neurons are particularly vulnerable to alterations in KP activity.

## 7. Conclusions

Accumulating experimental and preclinical evidence suggests that precise modulation of the kynurenine pathway (KP) may unlock a disease-modifying avenue that extends far beyond conventional therapies. The main goal of these therapies is to reduce the synthesis of toxic products and shift the balance toward metabolites that have a protective effect. Based on current preclinical and early clinical studies, it appears that the targets of KMO and IDO enzyme inhibitors, AHR receptor agonists, and KYNA analogues are the most clinically feasible in the near future. They may become a new and very important group of drugs that inhibit the neurodegeneration process in diseases such as Parkinson’s disease, Alzheimer’s disease, Huntington’s disease, and multiple sclerosis. 

Furthermore, both pharmacological and non-pharmacological modulation of KP also offers promising opportunities in the treatment of neurodegenerative diseases that have so far been treated only symptomatically. However, further, more detailed and interdisciplinary research combining immunology, neurobiology, pharmacology, and nutrigenomics is necessary to fully exploit the potential of KP-targeted treatment and, despite the challenges associated with its modulation, contribute to improving patients’ quality of life and providing effective and personalized therapeutic methods.

## Data Availability

No new data were created or analyzed in this study.

## Abbreviations

The following abbreviations are used in this manuscript:

1-MT	1-methyl-tryptophan
3-HANA	3-hydroxyanthranilic acid
3-HK	3-hydroxykynurenine
4-HNE	4-hydroxynonenal
6-OHDA	6-hydroxydopamine
7-Cl-KYNA	7-chlorokynurenate
AA	Anthranilic Acid
AchE	acetylcholinesterase
AhR	aryl hydrocarbon receptor
ALS	amyotrophic lateral sclerosis
ASC	caspase recruitment domain
BACE1	β-secretase
BBB	blood–brain barrier
BDNF	brain-derived neurotrophic factor
CNS	central nervous system
DAMP	damage-associated molecular patterns
EAE	experimental mouse model of autoimmune encephalomyelitis
FAD	flavin adenine dinucleotide
FDA	Food and Drug Administration
FMN	flavin mononucleotide
GDS	Global Deterioration Scale
HAA	3-hydroxyanthranilic acid
HFD	high-fat diet
IDO	indoleamine 2,3-dioxygenase
IL	interleukin
INF	interferon
KAT	kynurenine aminotransferases
KMO	kynurenine-3-monooxygenase;
KP	kynurenine pathway
KYNA	Kynurenic acid
KYNU	kynureninase
MAO	monoamine oxidase
MDA	malondialdehyde
MIA	maternal immune system
MTDLs	multi-targeted ligands
NAD	nicotinamide adenine dinucleotide
NLRP3	NOD-like receptor pyrin domain-containing 3
NMDA	N-methyl-D-aspartate
PAMP	pathogen-associated molecular patterns
PEG	percutaneous endoscopic gastrostomy
PGC-1α1	peroxisome proliferator-activated receptor gamma coactivator 1-α
PIC	Picolinic Acid
PLP	pyridoxal 5′-phosphate
PROB	organic acid transport inhibitor probenecid
PUFA	polyunsaturated fatty acids
QoL-AD	Quality of Life in Alzheimer’s Disease
QUIN	quinolinic acid
RNS	reactive nitrogen species
ROS	reactive oxygen species
TDO	tryptophan 2,3-dioxygenase
TLR	Toll-like receptor
TNF	Tumor necrosis factor
TRP	tryptophan
WHO-	World Health Organization
XA	Xanthurenic Acid
